# Unnatural Amino Acid: 4-Aminopyrazolonyl Amino Acid Comprising *Tri*-Peptides Forms Organogel With Co-Solvent (EtOAc:Hexane)

**DOI:** 10.3389/fchem.2022.821971

**Published:** 2022-05-05

**Authors:** Amarnath Bollu, Prajnanandan Giri, Nihar Ranjan Dalabehera, Asmita Rani Asmi, Nagendra K. Sharma

**Affiliations:** ^1^ National Institute of Science Education and Research (NISER), Bhubaneswar, India; ^2^ Homi Bhabha National Institute (HBNI), Mumbai, India

**Keywords:** ampyrone, aminopyrazolonyl amino acid, hybrid-β-peptides, organogelation, unnatural amino acid

## Abstract

Ampyrone is an amino-functionalized heterocyclic pyrazolone derivative that possesses therapeutic values such as analgesic, anti-inflammatory, and antipyretics. The chemical structure of ampyrone exhibits excellent hydrogen bonding sites and is considered as the potential scaffold of supramolecular self-assembly. Recently, this molecule has been derived into unnatural amino acids such as aminopyrazolone amino acid and its peptides. This report describes that one of its amino acids, *O*-alkylated ampyrone, containing hybrid (α/β) peptides forms organogel after sonication at 50–55°C with 0.7–0.9% (w/v) in ethyl acetate: hexane (1:3). The formation/morphology of such organogels is studied by nuclear magnetic resonance Fourier-transform infrared (FT-IR), circular dichroism (CD), scanning electron microscope (SEM), transmission electron microscopy (TEM), powder X-ray diffraction (Powder-XRD), and thermogravimetric analysis (TGA). Energy-minimized conformation of APA-peptides reveals the possibility of intermolecular hydrogen bonding. Hence, APA-peptides are promising peptidomimetics for the organogel-peptides.

## Graphical Abstract

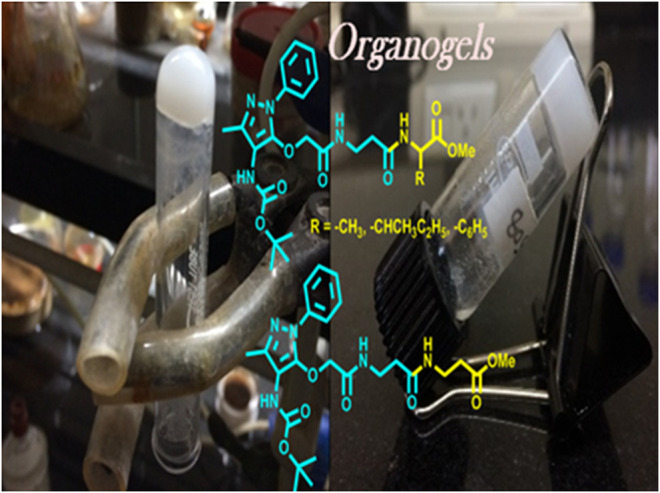

## Introduction

**FIGURE 1:**
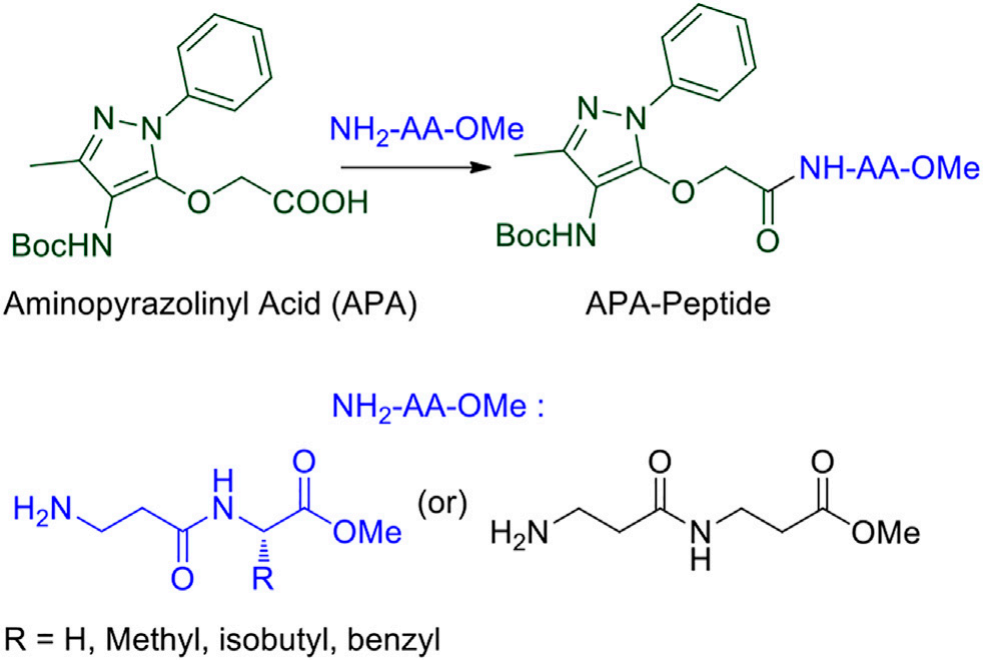
APA-appended peptides.

Peptides form self-assembly structures through non-covalent interactions, such as hydrogen bonding, van der Waals interactions, and π–π stacking (Zweep and Van Esch, 2013). The amide bonds and side chains of amino acid residues play a significant role in stabilizing the non-covalent interactions in peptides, which impart in the self-assembly of supramolecular structures including hydrogels and organogels (Hanabusa et al., 1993; Aggeli et al., 1997; Shaikh et al., 2018). Oligopeptides and small peptides are widely applied for the formation of versatile supramolecular organogels through these non-covalent interactions (Tomasini and Castellucci, 2013; Biswas et al., 2016). Sono-gels are a class of gels that are formed under ultrasound sonication and are widely applied for peptide-based gels (Li et al., 2007; Cravotto and Cintas, 2009). The process of gelation by ultrasound could involve breaking the larger aggregates or disordered aggregates to induce the formation of well-defined larger uniform aggregates which may lead to the formation of gels (Chatterjee and Maitra, 2017). The peptide-based organogels are biocompatible materials and considered promising biomaterials for various applications such as drug delivery (Couffin-Hoarau et al., 2004; Baral et al., 2014; Rouse et al., 2017), oil recovery in the petroleum industry (Chetia et al., 2020), and removal of toxic dyes (Li et al., 2020). Recently, the sequence-specific small peptides are explored to prepare thermally stable reversible/irreversible organogel biomaterials from natural/unnatural/hybrid peptides (Chakraborty et al., 2002; Banerjee et al., 2008; Maity et al., 2015; Wang and Yan, 2018). The insertion of aromatic structural unit/aromatic amino acid at the *N*-terminal of *di*-*tri*-peptides leads to the formation of stable organogel materials (Babu et al., 2014). 4-Aminopyrazole containing aromatic unnatural amino acids/dipeptides have abilities to interact with several bio-macromolecules such as interaction with specific β-sheet-rich targets in Aβ-protein and serine proteases via non-covalent interactions (Schrader and Kirsten, 1996; Kirsten and Schrader, 1997; Gilfillan et al., 2015; Hellmert et al., 2015). Previously, we have explored the structural and conformational studies of 4-aminopyrazolone amino acids/di-tri-peptide scaffold for non-covalent interactions, which is one of the important criteria for gelation (Bollu and Sharma, 2019b; a). We report the synthesis of 4-aminopyrazolone acid (APA) containing hybrid peptides with α-/β-amino acids and preparation of their *organogels* (Figure 1). These supramolecular self-assemblies are studied by NMR, FT-IR, CD, SEM, TEM, Powder-XRD, and TGA.

## Results and Discussion

**SCHEME 1:**
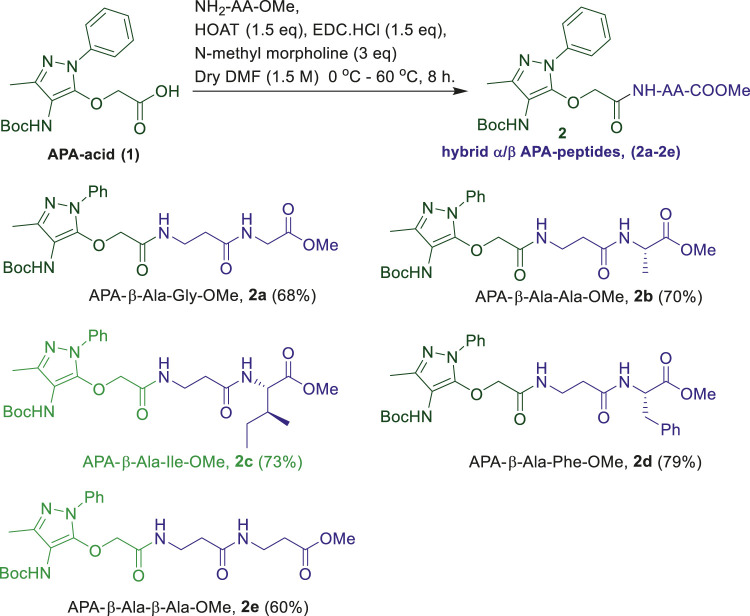
Synthesis of α-/β-hybrid APA-peptides.

We began the synthesis of the unnatural amino acid (**1**), aminopyrazolonyl acid (APA) by following the previously reported procedure (Scheme 1) (Bollu and Sharma, 2019b). In the literature, bipolar organic molecules have a higher propensity for organogelation (Bardelang et al., 2008; Loic, 2017). Thus, we planned to prepare bipolar molecules by introducing an APA unit at *N*-terminal of *di*-peptides containing hydrophobic side chain residues. We therefore synthesized APA *tri*-peptides (**2a**-**2e)** from α-β-hybrid peptide derivatives (NH_2_-AA-OMe) and APA (**1**). The hybrid peptides (NH_2_-AA-OMe) were prepared from β-alanine and α-amino acid (Gly/Ala/Ile/Phe). APA-*β*-Ala-Gly-OMe (**2a**) was prepared from dipeptide *β*-Ala-α-Gly-OMe, **2b** from *β*-Ala-α-Ala-OMe, **2c** from *β*-Ala-α-Ile, **2d** from *β*-Ala-α-Phe-OMe, and **2e** from β-Ala-*β*-Ala-OMe. These APA-peptides are well characterized by ^1^H-/^13^C-NMR/ESI-HRMS. Their respective spectra are provided in the Supplementary Material.

In the literature, the sequence-specific aromatic *tri-*peptides reportedly form organogel in the co-solvent systems (hexane:ethylacetate) after sonication (Maity et al., 2011; Maity et al., 2015). We also attempted the organogelation of unnatural aromatic amino acid, aminopyrazolonyl amino acid (APA), containing peptides (**2a-2e**) in the same co-solvent systems (hexane: ethylacetate) by sonication. The synthesized APA-peptides (**2a-2e)** 0.7–0.9% (wt/vol) were dissolved in solvent systems EtOAc:Hexane (1:3, v/v) and sonicated for 2 minutes above the room temperature (∼50°C) and then allowed to cool at room temperature. We noticed that the homogenous solutions of peptides (**2b-2e)** were transformed into colorless organogel within 10 min. However, the organogel formation was not noticed with hybrid APA-peptide **2a**. In the case of APA-peptide (**2d**), precipitation occurred at room temperature, however, upon heating converted into a homogenous solution. The hot homogenous solution was sonicated to form organogel within 2 minutes by allowing to cool at room temperature. In the literature, precipitates can also help in the formation of larger aggregates which can transform into gels (Li et al., 2007; Cravotto and Cintas, 2009). Importantly, the physical appearances of organogels of APA-peptides are different, such as transparent or opaque. We repeated a similar experiment with other solvents such as hexane, ethylacetate, benzene, chloroform, acetonitrile, and methanol but could not observe the gel formation. Mostly these peptides are sparingly soluble/or appeared as precipitates in those solvents. Moreover, for NMR studies, we attempted the organogelation of peptide **2e** in the deuterated solvent toluene-*d*_8_, and the formation of organogel was noticed after 2–3 days. The APA-peptide organogels are stable up to 50–55°C. At higher temperatures (above ∼55 °C), these gels are melted and eventually result in clear solutions. The formation of organogels was validated by the widely accepted inverted test tube method (Wang et al., 2003; Nagahama et al., 2008; Yoshida et al., 2014). Importantly, gelation is not observed when the β-Ala residue in APA-peptide is replaced with α-amino acid residues such as Gly, Ilu, and Ala (Bollu and Sharma, 2019b), indicating that the presence of β-Ala at that position is crucial for the formation of gels. Possibly, the presence of the β-Ala (extra methylene) group increases the chain length (extra–CH_2_-), which affects the intramolecular H-bonding interactions and flexibility in sol-state that can reorganize easily during the formation of the rigid gel networking aggregates, appearing as physical gels (Dado and Gellman, 1994; Roy et al., 2004; Chatterjee et al., 2007). However under similar conditions, compound **2a** (Gly residue at the C-terminal) did not form a gel.

### Morphology

**FIGURE 2:**
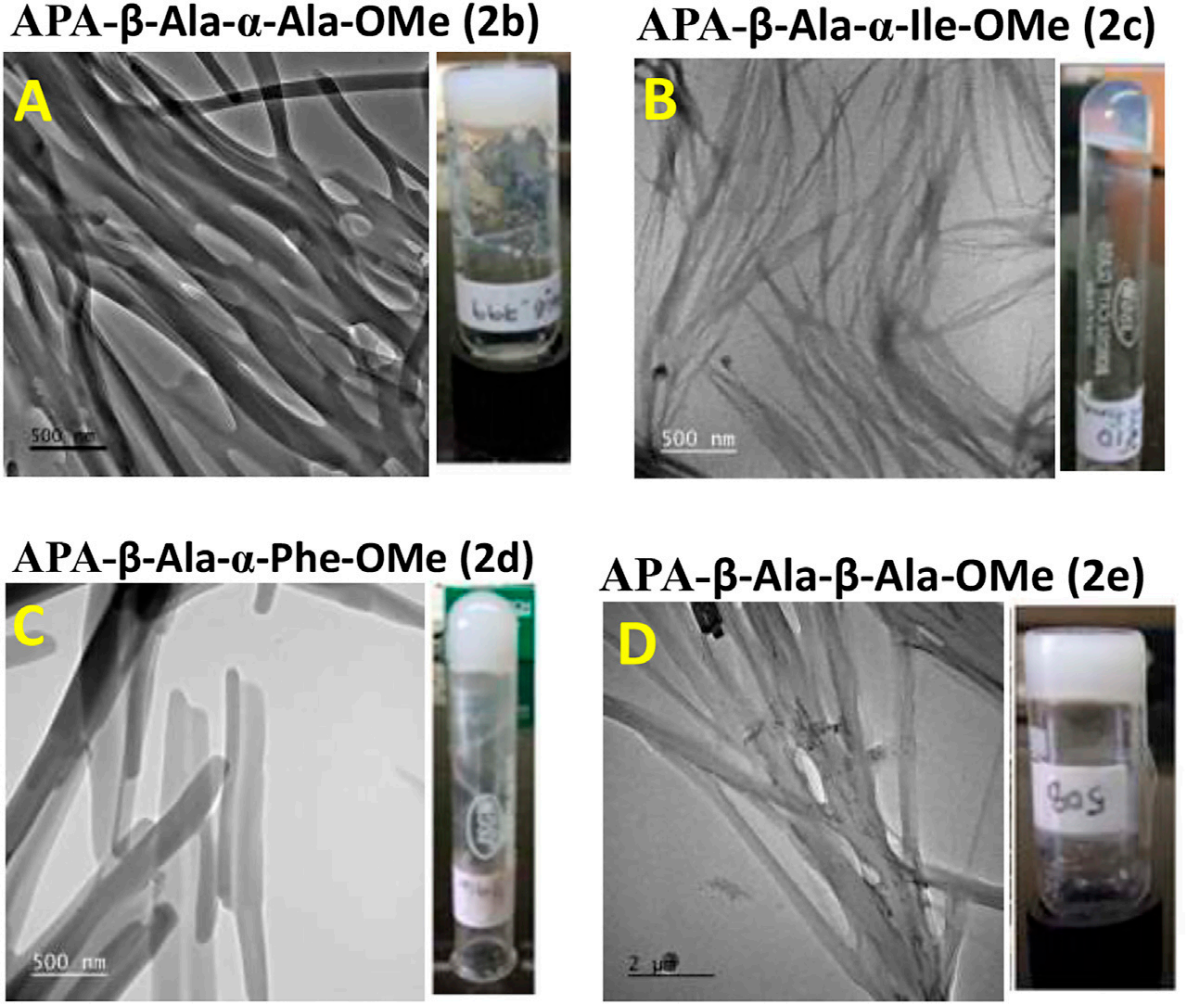
TEM image of APA-peptide organogels. Peptide APA-β-Ala-α-Ala-OMe, **2b (A)**; peptide APA-β-Ala-α-Ile-OMe, **2c (B)**; peptide APA-β-Ala-α-Phe-OMe, **2d (C)**; and peptide APA-β-Ala-β-Ala-OMe, **2e (D)**.

We studied the surface morphology of APA-peptide organogels by TEM (Transmission Electron Microscope) and SEM (Scanning Electron Microscope) imaging techniques. Their TEM images are depicted in Figure 2, while SEM images are provided in the SM (Supplementary Figure S20). We also inverted the sample vials containing APA-peptide organogel to confirm the formation of organogels (Figure 2). The TEM image of organogel of APA-peptide (**2b**) shows the formation of supramolecular self-assembly structure as a group of thick long linear fiber-forming complex structure (Figure 2A). The organogel of APA-peptide (**2c**) forms a supramolecular self-assembly structure as a thin short linear fiber structure (Figure 2B). The organogel of APA-peptide (**2d**) forms a supramolecular self-assembly structure as a small strip-type structure (Figure 2C). The organogel of APA-peptide (**2e**) forms a supramolecular self-assembly structure as a long rod-type structure (Figure 2D).

### Thermogravimetric Analysis

TGA of APA-peptides **2b-2e** in xerogel (dried organogel) and powder forms is measured with increasing temperature (with 5°C/min). (Haines, 1995). From TGA plots, we also extracted differential thermogravimetric (DTG) (Thürmer et al., 2014) plots (first order derivative plots), and all these plots are provided in the SM (Supplementary Figure S25). In xerogel and powder forms, weight loss from trapped solvent evaporation is observed below 100°C. In xerogel and powder form of peptides **2b-2d**, significant weight loss transitions are observed with two peaks between 200 and 300°C, whereas in APA-peptides **2e**, these weight loss transition peaks are observed at 170–225°C. Importantly, all APA-peptides in the xerogel form exhibit higher weight loss temperatures than the respective powder forms. Presumably, these weight loss peaks are either due to the loss of the sensitive Boc-protecting group or decompositions. These TGA and DTG plots demonstrate slightly enhanced stability of xerogels than their respective powder forms.

### UV Studies

We attempted to record the UV-vis spectra of APA-peptides (**2a-2e**) in polar/non-polar solvents. The UV-vis spectra of peptides **2a/2e** in MeOH exhibit an absorption peak at λ_245nm_ owing to the pyrazolone ring (Supplementary Material, Supplementary Figure S26). However, we were unable to record the UV spectra of peptides in ethylacetate and hexane owing to the poor solubility/precipitation.

### Circular Dichroism Studies

**FIGURE 3:**
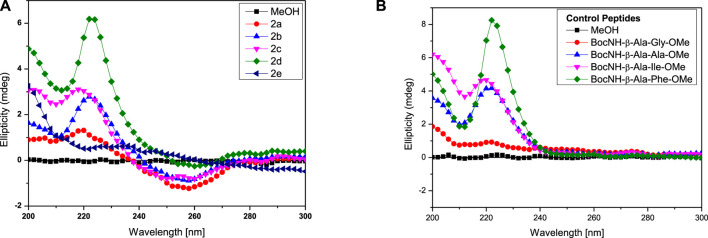
CD spectra of hybrid peptides in MeOH. APA-peptides **(A)** and control peptides **(B)**.

Circular Dichroism (CD) studies reveal the configuration and chirality of molecules including the nature of regular secondary structure (α-helix and β-strand) in protein, peptides, hydro-/organo-gels, and other chiral self-assembly materials (López Deber et al., 2014). However, the structure and conformation of peptides are sensitive to the nature of the solvent environment, which plays a significant role in peptides’ secondary structure formation (Cerpa et al., 1996; Awasthi et al., 2001). Previously, we have reported that the APA residue is involved in conformational changes of APA-peptides. We recorded the CD spectra of APA-peptides (**2a**–**2e**) of 0.1 mM concentrations in different solvent systems such as AcCN, MeOH, CHCl_3,_ and TFE. Their CD spectra in polar solvent MeOH are provided in Figure 3, while their CD spectra in other solvents are provided in the SM (Supplementary Figures S12–S14). For control studies, we also recorded the CD spectra of control peptides, without containing the APA-residue (Figure 3B, Supplementary Figures S15–S18). In MeOH solvent (polar protic), the CD spectra of APA-peptides (**2a**–**2e**) exhibit maxima at wavelength (*λ*) 220 nm (λ_220nm_) and minima at *λ*_260nm_. In contrast, the CD spectra of control peptides (in MeOH) exhibit only maxima at ∼λ_220nm_. The CD spectra of APA-peptides (**2a-2e**) exhibit almost similar CD signal maxima (λ_220nm_) and minima (λ_250nm_) in aprotic polar solvent acetonitrile (AcCN). However, the CD signals of APA-peptides (2a–2e) exhibit poorly resolved maxima and minima in solvent chloroform (CHCl_3_), and only maxima (*λ*_200nm_ and *λ*_220nm_) are observed in solvent trifluoroethanol (TFE). In the literature, TFE is well known to induce intramolecular hydrogen bonding which stabilizes possible helical structures, and such kind of CD structure is not observed with APA-peptides (**2a-2e**) (Sonnichsen et al., 1992). The CD signals of these peptides are possibly due to electronic transitions of the amide carbonyl group (π–π*/n–π*) at ∼λ_220nm_ and pyrazolonyl/phenyl aromatic rings (π–π*) at λ_250nm_. From the CD spectra of APA-peptides, overlapping of aromatic chromophoric (pyrazolonyl/phenyl) absorption (220–280 nm) with the finger print region of peptide secondary structure (190–240 nm) is observed. This made the interpretation of the secondary structures difficult. However, the maxima at ∼λ_220nm_ in APA-peptides (**2a-2e**) are presumed from the characteristics *β*-type of secondary structures (Maity et al., 2015).

**FIGURE 4:**
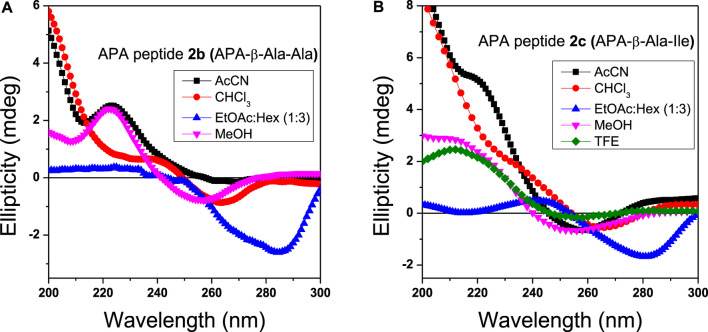
CD spectra of organogels **2b (A)** and **2c (B)** in different solvents (0.1 mM concentration). The CD spectra in ethylacetate and hexane mixture (blue) are significantly shifted.

We also studied the CD spectra of organogels of representative APA-peptides (**2b/2c**) in the co-solvent system EtOAc:Hexane (1:3, v/v) and other different polarity solvents such as AcCN, CHCl_3_, MeOH, and TFE (Figure 4). The CD spectra of APA-peptide organogels (**2b/2c**) in the co-solvent system EtOAc:Hexane (1:3, v/v) exhibit only minima at ∼λ_290nm_, and remarkable red-shift from λ_260nm_ strongly supports the existence of strong π–π interactions, possibly between two aromatic moieties (phenylpyrazolonyl unit) in organogel (Figures 4A,B). However the CD spectra of APA-peptides (**2b/2c**) in MeOH/TFE exhibit maxima (λ_260nm_) and minima (λ_260nm_). The CD spectra of that peptide organogel in other solvents are relatively non-characteristic. The solvent polarity and interaction of these solvents with APA-peptides resulted in diverse CD structures. The CD structures of APA-peptides in other solvents are presumed due to intermolecular H-bonding; this is further supported by our NMR and X-ray studies.

### NMR-Studies

**FIGURE 5:**
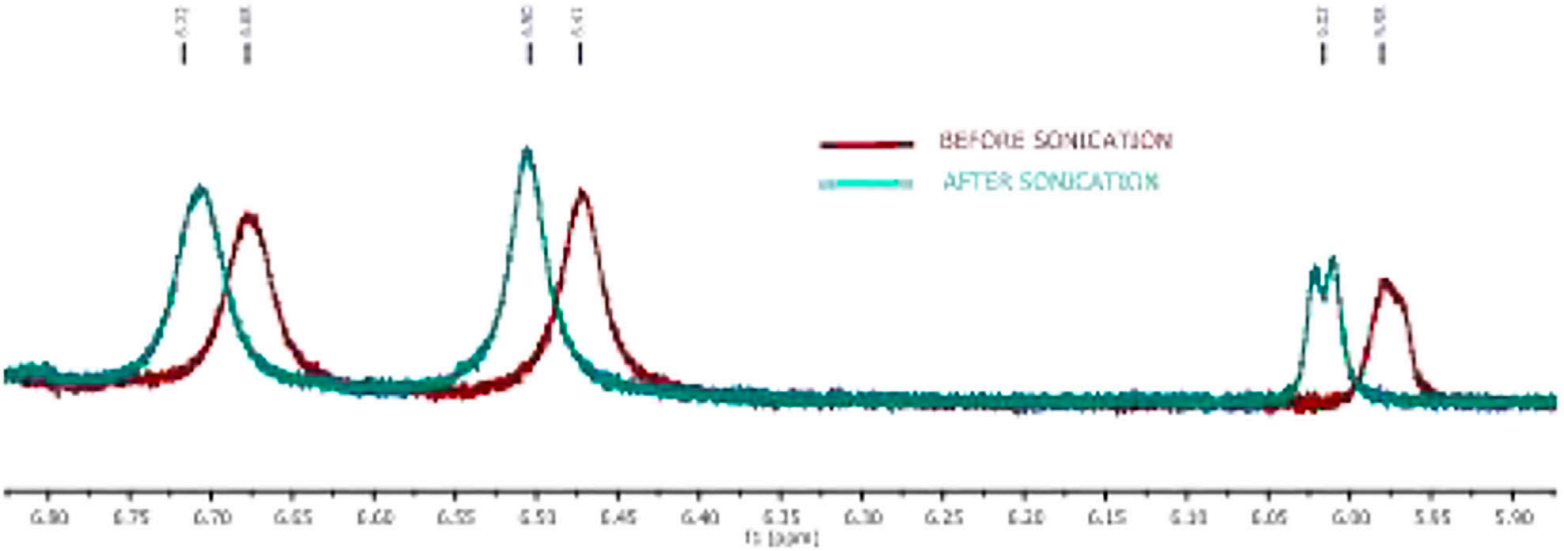
Expanded NMR of the peptide **2c** amide N-H region of organogel in toluene-d_8_ before (red) and after sonication (turquoise).

In the literature, the formation of peptide organogels is also studied by ^1^H-NMR in the deuterated solvent (toluene-*d*_8_) which exhibits a significant downfield chemical shift of amide N-H (Maity et al., 2015). We performed similar NMR studies with representative organogel-forming APA-peptide **2c** in the NMR solvent, toluene-d_8_ (Figure 5). The NMR spectra of amide N-H proton of peptide **2c** before/after organogelation are depicted in Figure 5 that exhibit the notable chemical shift in those amide N-H protons after sonication. This indicates that amide N-H is involved in hydrogen bonding after sonication that provides a relatively stronger hydrogen bonding environment. Similar NMR experiments were attempted with other organogel-forming peptides (**2b**/**2d**/**2e**) but were unable to record ^1^H-NMR in the same solvent, toluene-d_8_, because of instant solubility/precipitation.

APA-peptides (**2a-2e**) have three amide bonds which can involve in the hydrogen bonding network. We recorded 2D-NMR (^1^H-COSY) spectra for representative APA-peptide (**2c**) in CDCl_3_ and assigned their NH protons chemical shifts (δ) as Boc-NH (δ6.24), Ile-NH (δ6.41), and β-Ala-NH (δ7.31) (Supplementary Figure S22A). Notably, the β-Ala-NH is overlapped with aromatic protons; the cross-peaks in the ^1^H-COSY experiment are used to assign its chemical shift value. To study the amide bonds of APA-peptide (**2c**) involved in the hydrogen bonding network, we performed the ^1^H-NMR DMSO-d_6_ titration experiment in CDCl_3_ (Supplementary Figure S23)^.^(Malik et al., 2002; Balachandra and Sharma, 2014; Bollu and Sharma, 2019b) Since the amide bond (β-Ala-NH) appeared in the aromatic region, after DMSO-d_6_ titration, we again recorded 1H-COSY to confirm the respective amide NH (Supplementary Figure S22B). From these titration 1H-NMR spectra, with increasing concentration of DMSO-d_6_ (up to 19 μL), Boc-NH and Ile-NH exhibit a significant downfield shift; however, β-Ala-NH (appended at APA moiety) shows a marginal shift (Supplementary Figure S24). It appears that Boc-NH and Ile-NH are involved in intermolecular hydrogen bonding, and β-Ala-NH is involved in intramolecular hydrogen bonding for the formation of the secondary structure.

### FT-IR Studies of Organogels

**FIGURE 6:**
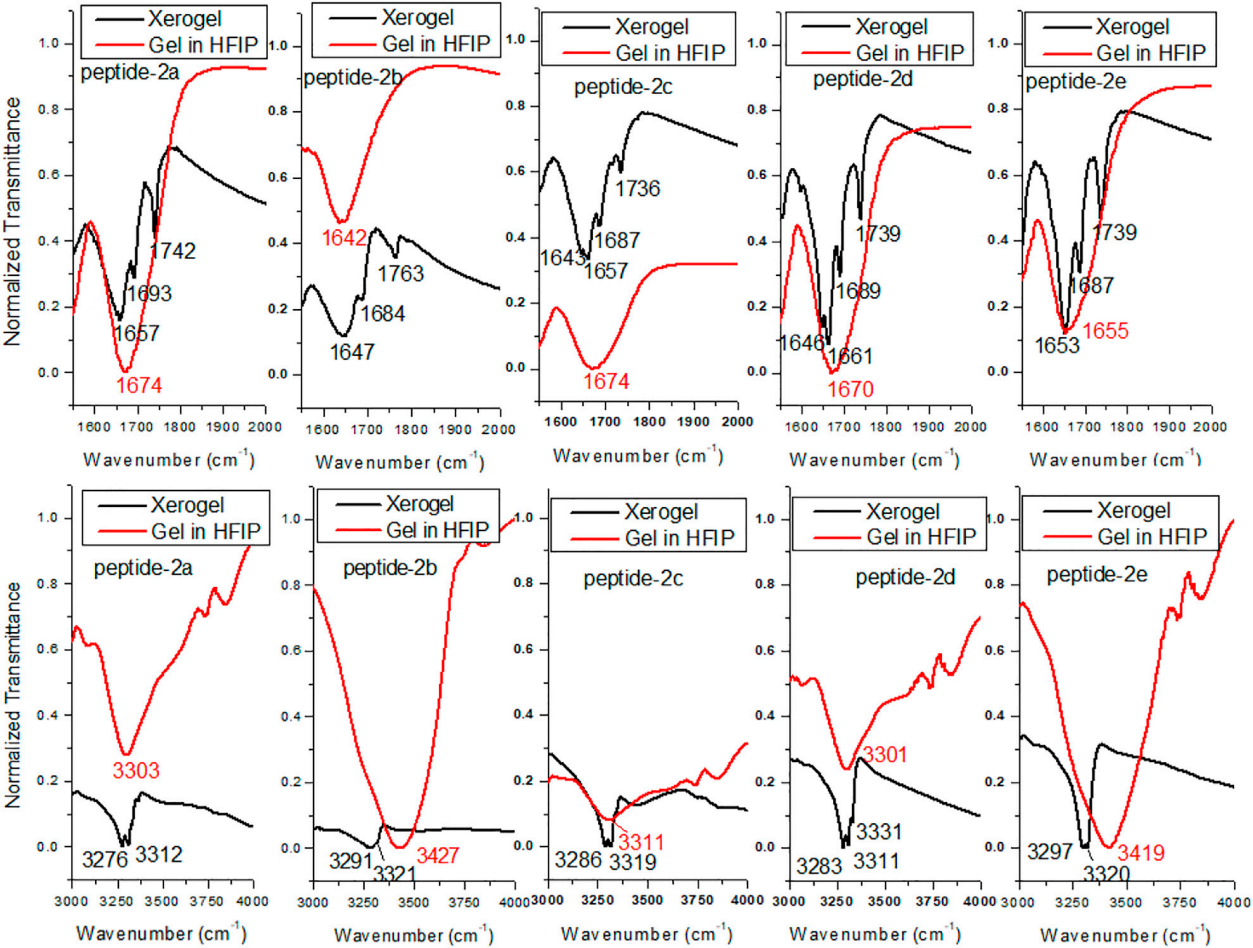
FT-IR of APA-peptide **2a**-**2e** xerogels and gels dissolved in HFIP. Carbonyl region (top) and amide NH region (bottom).

FT-IR spectral analyses also support the formation of organogel in the sequence-specific peptides (Malik et al., 2002; Maity et al., 2015). It is reported that the IR frequency of free N-H stretching (Amide-A band) appears at ∼3400 cm^−1^, while hydrogen-bonded N-H appears at a lower frequency of ∼3300 cm^−1^s (Vass et al., 2003; Adochitei and Drochioiu, 2011). Also, the frequency of free amide-1 band (C=O stretching vibration) appears at 1680 cm^−1^, while hydrogen-bonded C=O vibration appears at lower frequency ∼1650 cm^−1^ in organogels/xerogels (Bardelang et al., 2008; Maity et al., 2015). Importantly, IR peaks in organogel/xerogel are more structured than those in synthesized peptides. To prevent the self-aggregations through intermolecular hydrogen bonding, we planned to record the IR spectra of APA-peptides (**2a-2e**) organogel in hexafluoroisopropanol (HFIP) solvent. Thus, we recorded the FT-IR spectra of clear xerogels of APA-peptides (**2a-2e**) and compared with IR spectra of organogel in HFIP solvent. Their carbonyl and amide region spectra are depicted in Figure 6, while their whole spectra are provided in the SM (Supplementary Figure S19). The FT-IR spectra of clear xerogels of APA-peptides (**2a**-**2e**) exhibit resolved peaks at ∼1,645–1,657 cm^−1^, ∼1,684–1,693 cm^−1^, and ∼1736–1763 cm^−1^ which belong to the stretching frequency of the amide carbonyl, carbamate carbonyl, and ester carbonyl, respectively. However, the FT-IR spectra of those organogels in HFIP solvent exhibit a non-resolved broad peak at 1674 cm^−1^. We also found that the N-H (Amide-A) stretching vibrations appear at ∼3,276–3,312 cm^−1^, which is lower than free N-H stretching frequency (∼3400 cm^−1^). In the literature, the *β*-sheet-forming peptides show amide-1 (amide carbonyl) stretching frequency at ∼1625–1650 cm^−1^, while α-helix-forming peptides at ∼1,650–1,660 cm^−1^ that is lower than free amide carbonyl stretching frequency (∼1680 cm^−1^ (Vass et al., 2003; Yuran et al., 2012). The FT-IR spectra of other xerogel peptides/organogel in HFIP are almost same. Thus, our FT-IR spectral analyses support the formation of secondary structure as α-helix/β-sheet types in xerogel (**2a**-**2e**).

### X-Ray Diffraction Analysis

**FIGURE 7:**
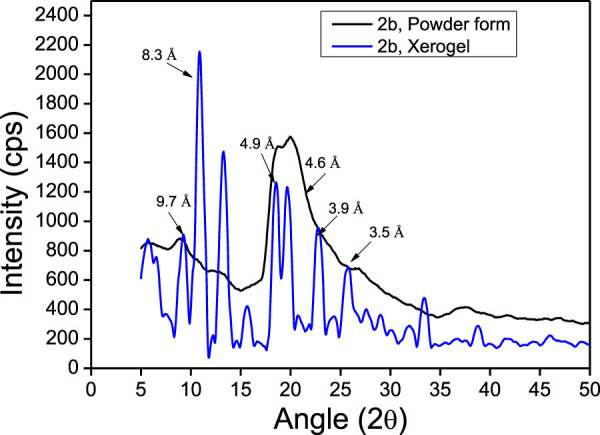
Powdered XRD reflection pattern of APA-peptide **2b**.

The powder X-ray diffraction studies are used to confirm the supramolecular self-assembly structure in xerogels including peptide-based xerogels (dry organogels) (Marchesan et al., 2012; Marchesan et al., 2014). A typical peptide xerogel exhibit sharp reflection peaks at 5–35° 2θ (reflection angle) range, while non-xerogel peptides (synthetic) exhibit broad reflection peaks at 20° 2θ range. We also performed a powder X-ray diffraction experiment with organogel-forming APA-peptides (**2b-2c**). We recorded the X-ray diffraction (XRD) spectra of peptides **2b-2e** in powder form (before organogelation) and their respective xerogel. The XRD spectra of the APA-peptide (**2b**) solid powder (before/after gelation) are depicted in Figure 7, while for other APA-peptides (**2c-2e**) are provided in the SM (Supplementary Figure S21). The XRD spectra of peptide (**2b**) show that its xerogel powders are structurally organized than the powder before gelation. We calculated d-spacing values in angstroms (Å) from their experimental 2θ reflection peaks by applying Bragg’s equation (nλ = 2dSinθ) (Bardelang et al., 2008; Marchesan et al., 2012). Their d-spacing values are provided above the reflection peaks. In xerogel spectra, the reflection peaks at 4.5–5.1Å are characteristics for hydrogen bonding between β-strands, while peaks at 9.7–10.8Å are associated with the distance between anti-parallel strands (i.e. every other strand) or to inter-sheet distances. The peaks at 3.8–4.2Å are attributed to π-π stacking possible from aromatic N-phenyl pyrazole rings (Marchesan et al., 2012; Marchesan et al., 2014). Thus, APA-peptide xerogels have the β-sheet type of structure in their supramolecular self-assembly structure.

### Conformational Studies

**FIGURE 8:**
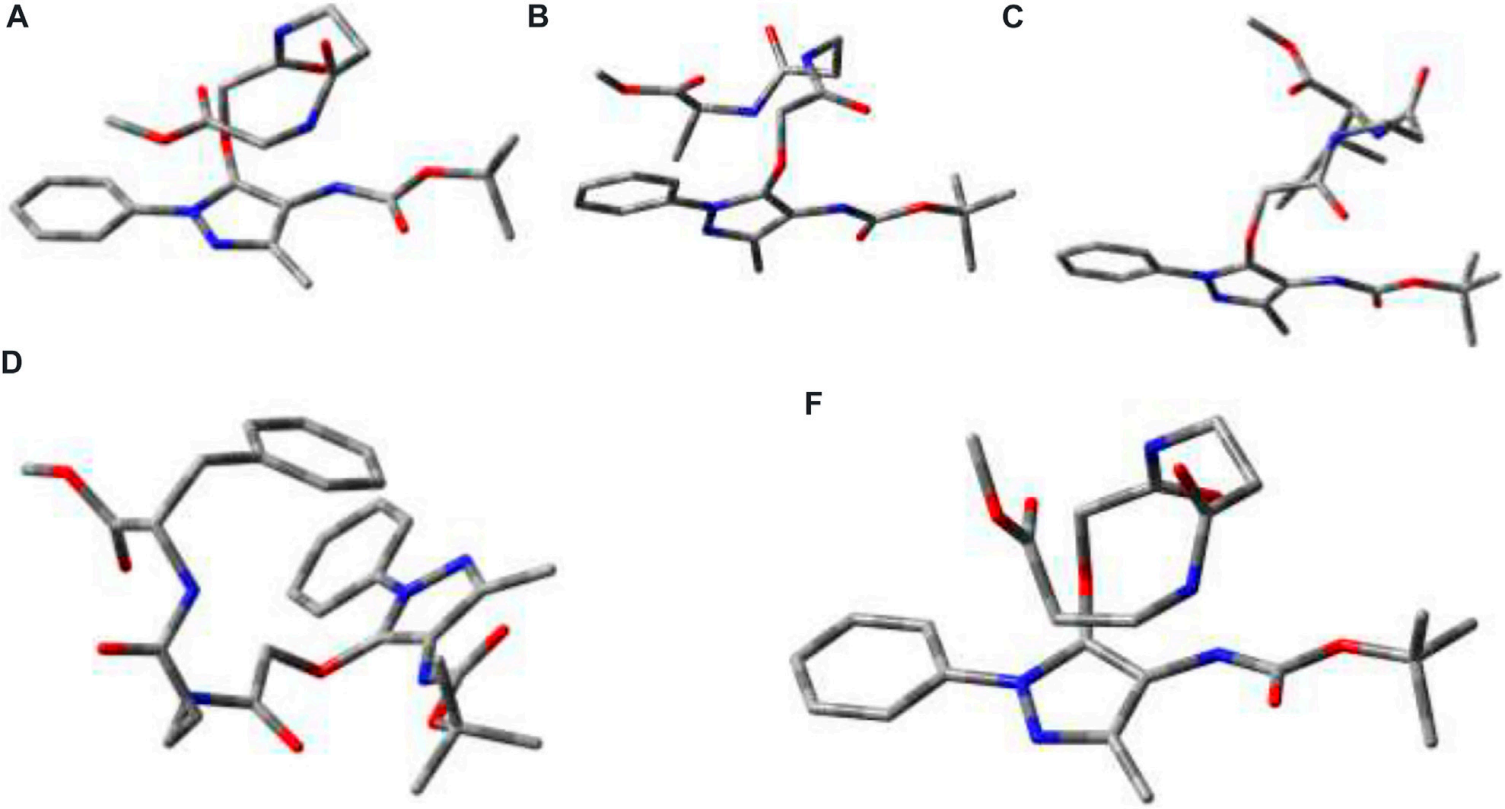
Energy-minimized conformer without hydrogen atoms. **2a** (*E* = 31.2 kcal/mol; GBSA steric energy = 18.6 kca/mol; dielectric constant: 1; dipole moment: 4.2); **2b** (*E* = 34.7 kcal/mol; GBSA steric energy = 22.3 kca/mol; dielectric constant: 1; dipole moment: 4.2); **2c** (*E* = 38.8 kcal/mol; GBSA steric energy = 25.8 kcal/mol; dielectric constant: 1; dipole moment: 5.0); and **2d** (*E* = 56.0 kcal/mol; GBSA steric energy = 41.4 kcal/mol; dielectric constant: 1; dipole moment: 6.0); **2e** (*E* = −3.0 kca/mol; GBSA steric energy = −15.6 kca/mol; dielectric constant: 1; dipole moment: 3.1).

Global-MMX (GMMX) is a steric energy minimization program that uses the supported force field (MMX, MM3, or MMFF94) and operates in batch mode to search conformational space and to list the lowest energy unique conformers. The generalized born/surface area (GB/SA) solvation model gives free energies of aqueous solvation (Cheng et al., 2000). GMMX and GBSA solvation calculation models are being frequently applied to find the energy-minimized conformation of peptides in the gas phase and water medium (Lee et al., 2001; Biswas et al., 2013). We performed the theoretical calculation to find the energy-minimized conformation into the gas phase and solution with GMMX and GBSA solvation methods with the MMFF94 force field. The details are proved in the Supplementary Material. The structurally minimized conformers of APA-peptides (**2a-2e**) without hydrogen atoms are provided in Figure 8 while with hydrogen atoms in Supplementary Figure S27. The stabilization energy of APA-peptide (**2a-2e**) solution phase (dielectric constant, equivalent to water) is lower than that of the gas phase by 12∼kcal/mol without affecting the significant changes in structural conformation. Importantly, we could not find *intramolecular* hydrogen bonding in the energy-minimized conformers of APA-peptides. Their phenyl-aminopyrazolone residues are planar, and the polar groups are exposed in solvents which could participate in the intermolecular hydrogen bonding with other molecules. Generally, intramolecular hydrogen bonding prevents the formation of organogels. Presumably, these APA-peptides form *intermolecular* hydrogen bonding in the organic solvent system (EtOAc:Hexane) after sonication and produce organogels. Hence, APA-peptides have the ability to form organogel.

## Conclusion

Aminopyrazolonyl amino acid (APA) containing α-/β-hybrid peptides are explored further for supramolecular self-assembly structure by the formation of organogel in the organic solvent system. Most of them form organogels, but their physical appearances are different such as opaque and translucent. These organogels are characterized as β-sheet types of the structure by NMR, IR, CD, powder-XRD, TGA, SEM, and TEM techniques. Theoretically, the energy-minimized structure suggests that there is no *intra*molecular hydrogen bonding in the polar solvent. There could be possibility of the formation of intermolecular hydrogen bonding after sonication in the organic solvent which leads to the formation of organogel in the EtOAc:Hexane solvent system. Hence, the APA acid could be employed at the *N*-terminal of target di-/tri-peptides for organogelation in the organic co-solvent (EtOAc:Hexane) system.

## Experimental Details

### Materials

All required materials were obtained from commercial suppliers and used without any further purification. Dimethylformamide was distilled with calcium hydride. Reactions were monitored by TLC (thin layer chromatography) and visualized by UV and ninhydrin. Column chromatography was performed in a 230–400 mesh silica gel. Mass spectra and HRMS were obtained using the Bruker micrOTOF-Q II spectrometer. ^1^H NMR and ^13^C NMR were recorded on Bruker AV-400 or 700 MHz at 298 K. ^1^H and ^13^C NMR chemical shifts were recorded in ppm downfield from tetramethylsilane or residual solvent peak. Splitting patterns are abbreviated as follows: s, singlet; d, doublet; dd, doublet of doublet; t, triplet; q, quartet; dq, doublet of the quartet; and m, multiplet. Powder X-ray diffraction data were collected on a Bruker D8 Advance with DA VINCI design fitted with an HTK 16 temperature chamber X-ray powder diffractometer using CuKα radiation (*λ* = 1.5418 Å). Transmission electron microscopy (TEM) data were recorded using JEOL 2100F.

### General Experimental Procedure for Compounds (2a-2e)

The experimental procedures for the synthesis of control dipeptides and APA-peptides (**2a-2e**) were followed from the literature. (Bollu and Sharma, 2019b).

*APA-β-Ala-Gly-OMe* (2a). *R*_*f*_ 0.18 (0.4:9.6 MeOH/CH_2_Cl_2_); yield 68%; ^1^H NMR (400 MHz, DMSO) δ 8.37 (s, 1H), 8.17 (s, 1H), 8.05 (s, 1H), 7.71 (d, *J* = 7.5 Hz, 2H), 7.46 (t, *J* = 7.6 Hz, 2H), 7.30 (t, *J* = 7.1 Hz, 1H), 4.60 (s, 2H), 3.83 (d, *J* = 5.5 Hz, 2H), 3.61 (s, 3H), 3.32 (s, 2H), 2.36 (t, *J* = 6.9 Hz, 2H), 2.03 (s, 3H), and 1.44 (s, 9H); ^13^C NMR (176 MHz, DMSO) δ 171.56, 170.95, 167.16, 162.16, 155.30, 147.65, 146.78, 138.90, 129.55, 126.76, 122.28, 103.74, 79.39, 70.54, 52.22, 45.65, 41.05, 35.63, 35.22, 28.61, and 12.40. HRMS (ESI-TOF) m/z [M + H]^+^ Calcd for C_23_H_31_N_5_O_7_ 490.2296; found 490.2295.

***A*** *PA-β-Ala-Ala-OMe* (2b). *R*_*f*_ 0.33 (0.4:9.6 MeOH/CH_2_Cl_2_); yield 70%; ^1^H NMR (400 MHz, DMSO) δ 8.35 (d, *J* = 6.9 Hz, 1H), 8.17 (s, 1H), 8.04 (s, 1H), 7.70 (d, *J* = 7.8 Hz, 2H), 7.46 (t, *J* = 7.9 Hz, 2H), 7.30 (t, *J* = 7.3 Hz, 1H), 4.60 (s, 2H), 4.26 (p, *J* = 7.2 Hz, 1H), 3.61 (d, *J* = 5.4 Hz, 3H), 3.40–3.21 (m, 3H), 2.39–2.27 (m, 2H), 2.02 (s, 3H), 1.43 (s, 9H), 1.25 (d, *J* = 7.3 Hz, 4H). ^13^C NMR (176 MHz, DMSO) δ 173.75, 170.98, 167.17, 155.32, 147.66, 146.80, 138.93, 129.58, 126.79, 122.30, 103.77, 79.41, 70.55, 52.40, 48.06, 35.63, 35.20, 28.64, 17.49, and 12.43. HRMS (ESI-TOF) m/z [M + Na]^+^ Calcd for C_24_H_33_N_5_O_7_Na 526.2272; found 526.2272.

*APA-β-Ala-Ile-OMe* (2c). *R*_*f*_ 0.35 (0.3:9.7 MeOH/CH_2_Cl_2_); yield 73%; ^1^H NMR (400 MHz, CDCl_3_) δ 7.61 (d, *J* = 7.9 Hz, 2H), 7.43 (t, *J* = 7.7 Hz, 2H), 7.37–7.24 (m, 1H), 6.34 (d, *J* = 72.2 Hz, 1H), 4.57 (m, *J* = 41.5, 26.2 Hz, 3H), 3.67 (d, *J* = 27.7 Hz, 4H), 3.51 (s, 1H), 2.48 (d, *J* = 4.8 Hz, 2H), 2.17 (s, 3H), 1.81 (s, 1H), 1.48 (s, 9H), 0.89 (d, *J* = 6.0 Hz, 6H). ^13^C NMR (101 MHz, CDCl_3_) δ 172.81, 172.04, 167.72, 155.29, 147.54, 146.92, 138.24, 129.04, 126.72, 122.38, 102.45, 80.59, 70.89, 56.91, 52.08, 37.06, 35.57, 28.25, 25.25, 15.50, 11.87, and 11.40. HRMS (ESI-TOF) m/z [M + H]^+^ Calcd for C_27_H_40_N_5_O_7_ 546.2933; found 546.2812.

*APA-β-Ala-Phe-OMe* (2d). *R*_*f*_ 0.36 (0.4:9.6 MeOH/CH_2_Cl_2_); yield 79%; ^1^H NMR (400 MHz, DMSO) δ 8.42 (d, *J* = 7.7 Hz, 1H), 8.16 (s, 1H), 7.98 (s, 1H), 7.69 (d, *J* = 7.7 Hz, 2H), 7.45 (t, *J* = 7.7 Hz, 2H), 7.33–7.16 (m, 7H), 4.58 (s, 2H), 4.47 (dd, *J* = 13.9, 8.2 Hz, 1H), 3.60 (s, 3 Hz), 3.22 (dd, *J* = 20.2, 10.2 Hz, 2H), 3.01 (dd, *J* = 13.6, 5.3 Hz, 1H), 2.88 (dd, *J* = 13.4, 9.5 Hz, 1H), 2.30 (m, *J* = 14.6, 7.4 Hz, 2H), 2.02 (s, 3H), 1.43 (s, 9H). ^13^C NMR (176 MHz, DMSO) δ 172.66, 172.57, 171.15, 170.40, 167.16, 155.33, 147.67, 146.81, 138.90, 137.77, 129.59, 128.80, 127.13, 126.81, 122.32, 103.72, 79.43, 70.53, 54.12, 52.41, 45.69, 37.24, 36.33, 35.58, 35.21, 34.29, and 28.63, 12.42. HRMS (ESI-TOF) m/z [M + H]^+^ Calcd for C_30_H_38_N_5_O_7_ 580.2766; found 580.2760.

APA-β-Ala-β-Ala-OMe (2e). *R*_*f*_ 0.28 (0.3:9.7 MeOH/CH_2_Cl_2_); yield 60%; ^1^H NMR (400 MHz, CDCl_3_) δ 7.56 (d, *J* = 7.6 Hz, 2H), 7.41 (t, *J* = 7.7 Hz, 2H), 7.36–7.14 (m, 2H), 6.45 (s, 1H), 6.35 (s, 1H), 4.58 (s, 2H), 3.66 (s, 3H), 3.60–3.43 (m, 2H), 2.44 (d, *J* = 40.0 Hz, 2H), 2.18 (s, 3H), 1.47 (s, 9H). HRMS (ESI-TOF) m/z [M + H]^+^ Calcd for C_24_H_34_N_5_O_7_ 504.2453; found 504.2453.

#### Organogelation

A measure of 10 mg of APA-peptides (**2a-2e)** was dissolved in 1 ml of hexane–ethylacetate (3:1) solvent mixture and sonicated at 50°C for 2 min and then allowed to cool at room temperature. Under these conditions, APA-peptides (**2b-2e**) formed organogels.

#### Field Emission Scanning Electron Microscopy

A measure of 10 mg of APA-peptides (**2b-2e)** was dissolved in 1 ml of hexane–ethylacetate (3:1) mixture and sonicated at 50°C for 2 min. Then, the gel was casted on the silicon wafer and dried under high vacuum, and SEM images were obtained at 3.00 kV.

#### Field Emission Transmission Electron Microscopy

A measure of 10 mg of APA-peptides (**2b-2e)** was dissolved in 1 ml of hexane–ethylacetate (3:1) mixture and sonicated at 50°C for 2 min. Then, the gel was diluted 3–4 times and casted on a copper grid and dried under high vacuum; TEM images were obtained.

#### Circular Dichroism Spectroscopy

CD spectra were recorded in degassed CH_3_OH, AcCN, CHCl_3_, CF_3_CH_2_OH, and hexane–ethylacetate (3:1) at 20°C from 300–200 nm with peptide concentrations of 0.1 mM. CD data are collected with following parameters: data pitch at 2 nm, DIT for 2 s, bandwidth at 2 nm, and scanning speed at 100 nm/min.

#### Fourier-Transform Infrared Spectroscopy

Peptide gels were drop-casted on the KBr window and dried under high vacuum. For HFIP, xerogels were dissolved in HFIP and drop-casted on the KBr window and then dried under high vacuum. The spectra are the average of 250 scans.

## Data Availability

The datasets presented in this study can be found in online repositories. The names of the repository/repositories and accession number(s) can be found in the article/Supplementary Material.

## Abbreviations

APA 4, aminopyrazolone acid.
