# Supplementary material for: Unnatural Amino Acid: 4-Aminopyrazolonyl Amino Acid Comprising Tri-Peptides Forms Organogel With Co-Solvent (EtOAc:Hexane)
Source: Front Chem. 2022 May 5;10:821971. doi: 10.3389/fchem.2022.821971 (PMC9117720; doi:10.3389/fchem.2022.821971)
Supplement: Supplementary file 1 [file DataSheet1.pdf]

Supplementary Material (SM)

Unnatural Amino Acid: 4-Aminopyrazolonyl Amino acid Comprising *tri*-Peptides Form Organogel with Co-solvent (EtOAc:Hexane)

Amarnath Bollu,[a,b] Prajnanandan Giri,[a,b] Nihar Ranjan Dalabehera,[a,b] Asmita Rani Asmi,[a,b] Nagendra K Sharma* [a,b]

[a]National Institute of Science Education and Research (NISER)-Bhubaneswar, Jatni Campus, Jatni-752050 (Odisha)-India;

[b]Homi Bhabha National Institute (HBNI), HBNI-Mumbai, Mumbai, 400 094, India.

Contents

1. ^{1}H-/^{13}C-/ ESI-MS/HRMS spectra of 2a S2
2. ^{1}H-/^{13}C-/ ESI-MS/HRMS spectra of 2b S4
3. ^{1}H-/^{13}C-/ ESI-MS/HRMS spectra of 2c S6
4. ^{1}H-/^{13}C-/ ESI-MS/HRMS spectra of 2d S8
5. ^{1}H-/^{13}C-/ ESI-MS/HRMS spectra of 2e S10
6. Circular Dichroism (CD) spectra control peptides and 2a-2e. S12
7. FT-IR spectra of peptide organogel (2b/2c/2d/2e) S16
8. SEM Images of peptide organogel (2b/2c/2d/2e) S17
9. Powder XRD of peptides 2b/2c/2d/2e. S18
10. ^{1}H-COSY- NMR and DMSO-d6 titration S19
11. TGA and Derivative TGA plots of peptides 2b/2c/2d/2e. S22
12. UV-Vis Spectra of peptides 2c/2e in MeOH. S24
13. Theoretical calculation of APA-peptides conformers S24
14. Reference S26

1. ^{1}H-/^{13}C-/ ESI-MS/HRMS spectra of **2a**

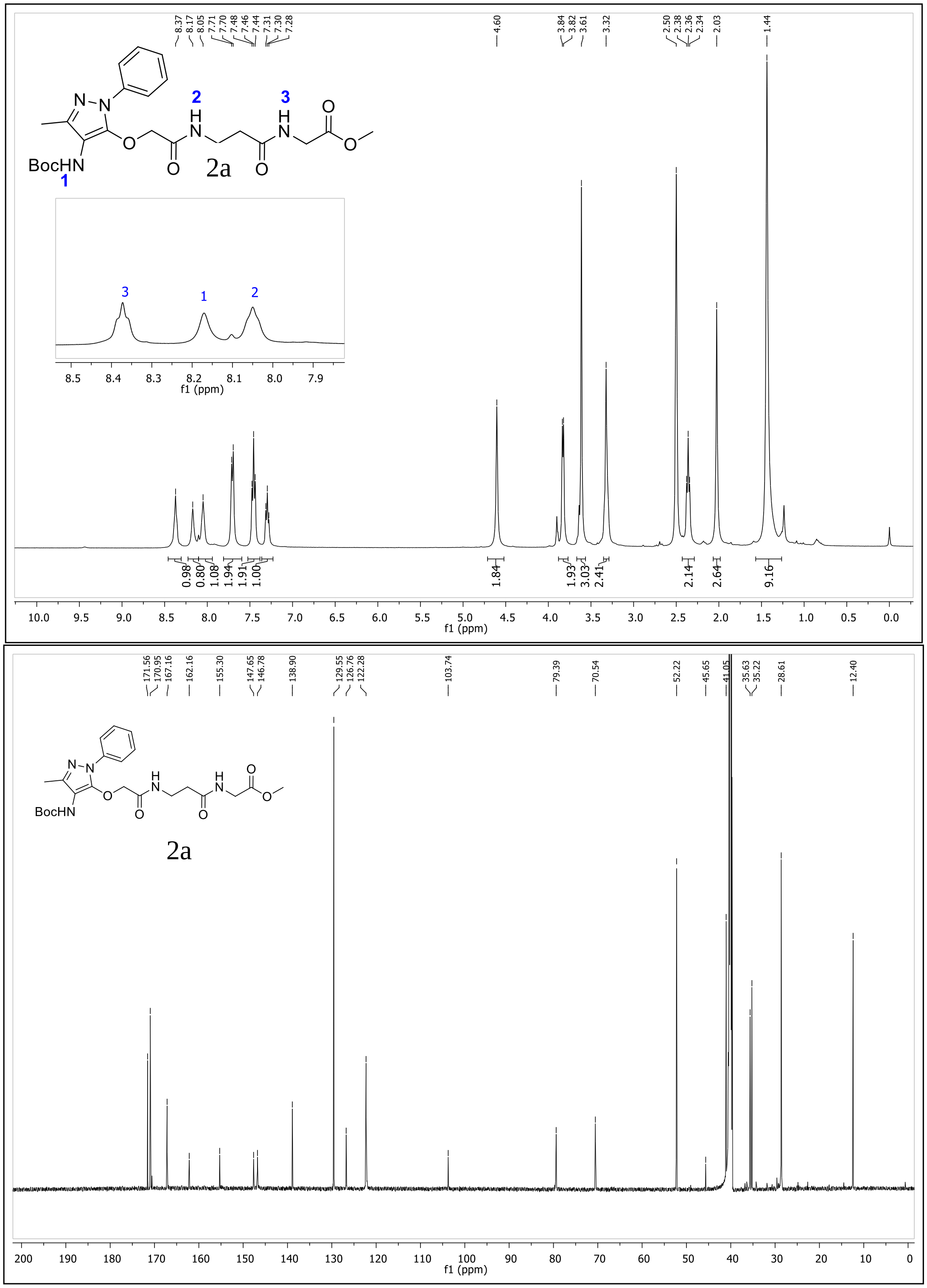

Figure S1. ^{1}H-NMR (400MHz) and ^{13}C-NMR (176MHz) spectra of **2a** in DMSO-d6

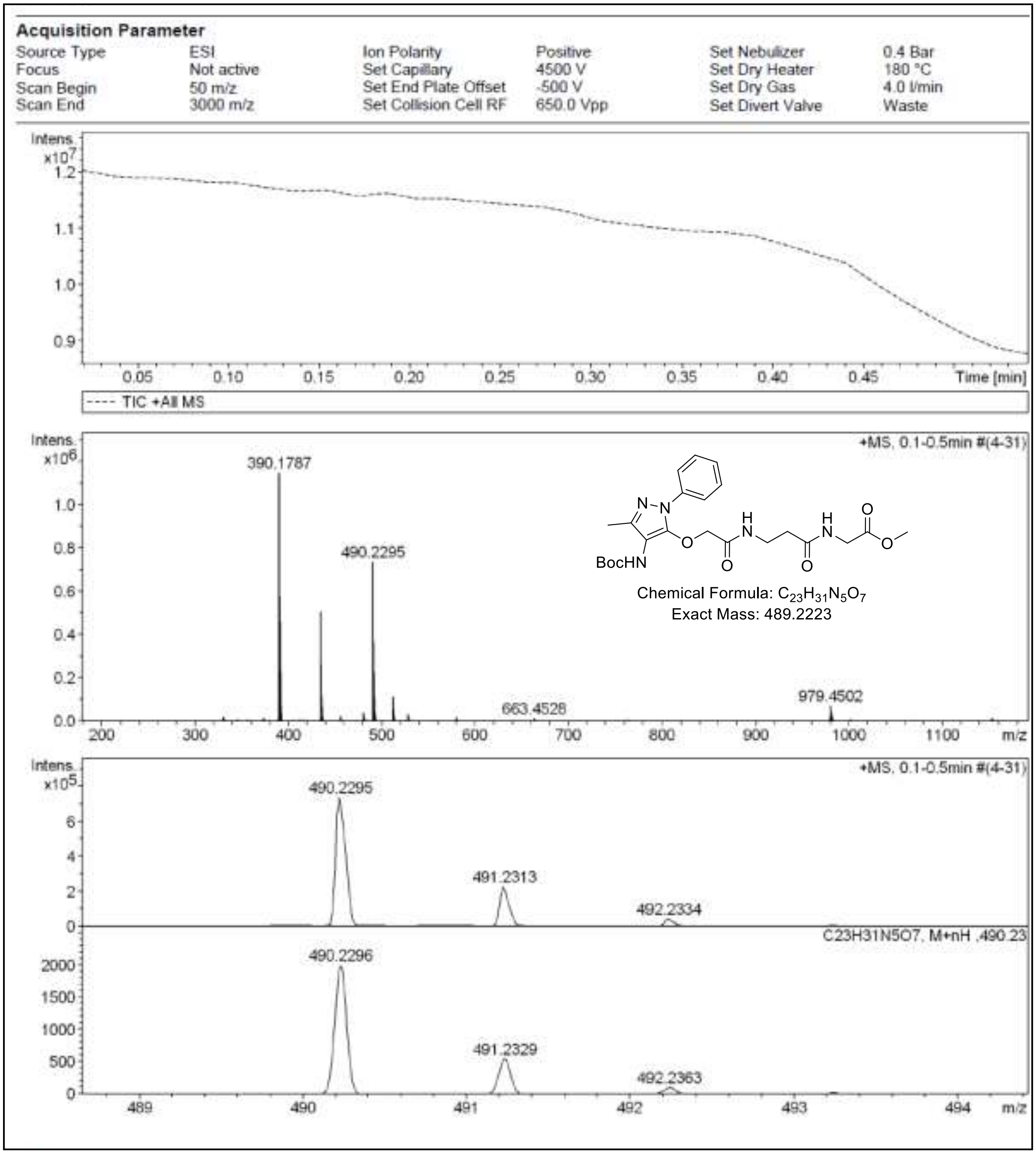

Figure S2. ESI-MS/HRMS spectra of **2a**

2. ^{1}H-/^{13}C-/ ESI-MS/HRMS spectra of **2b**

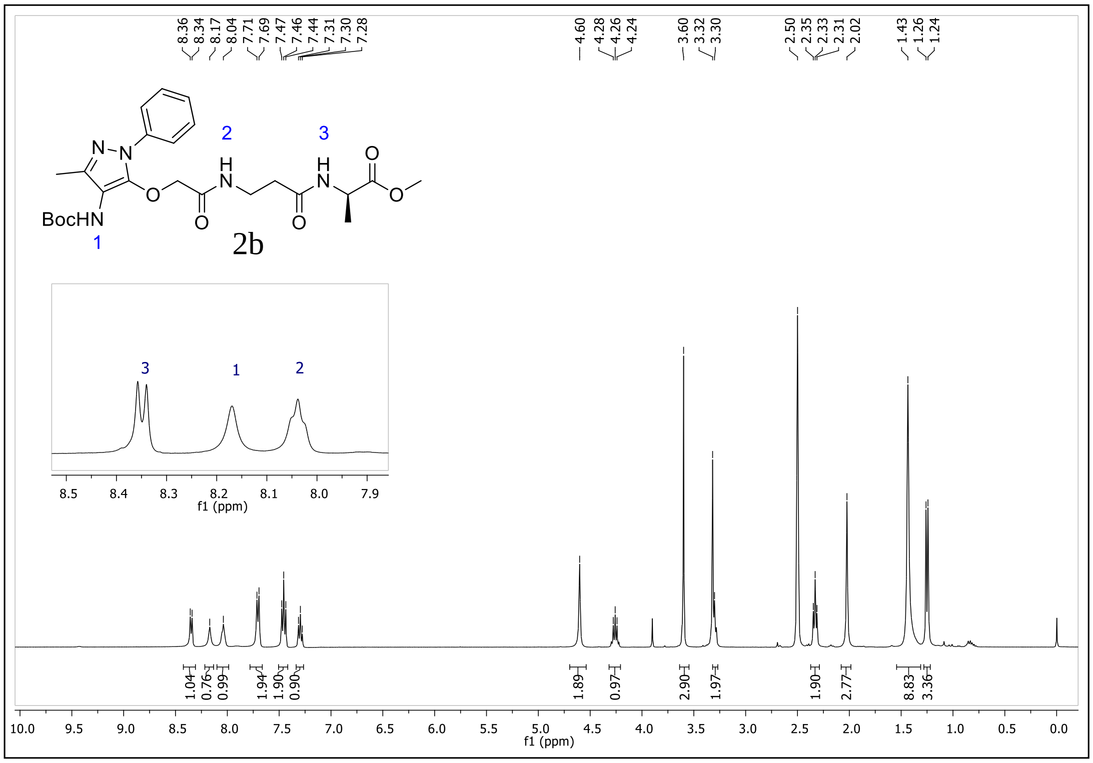

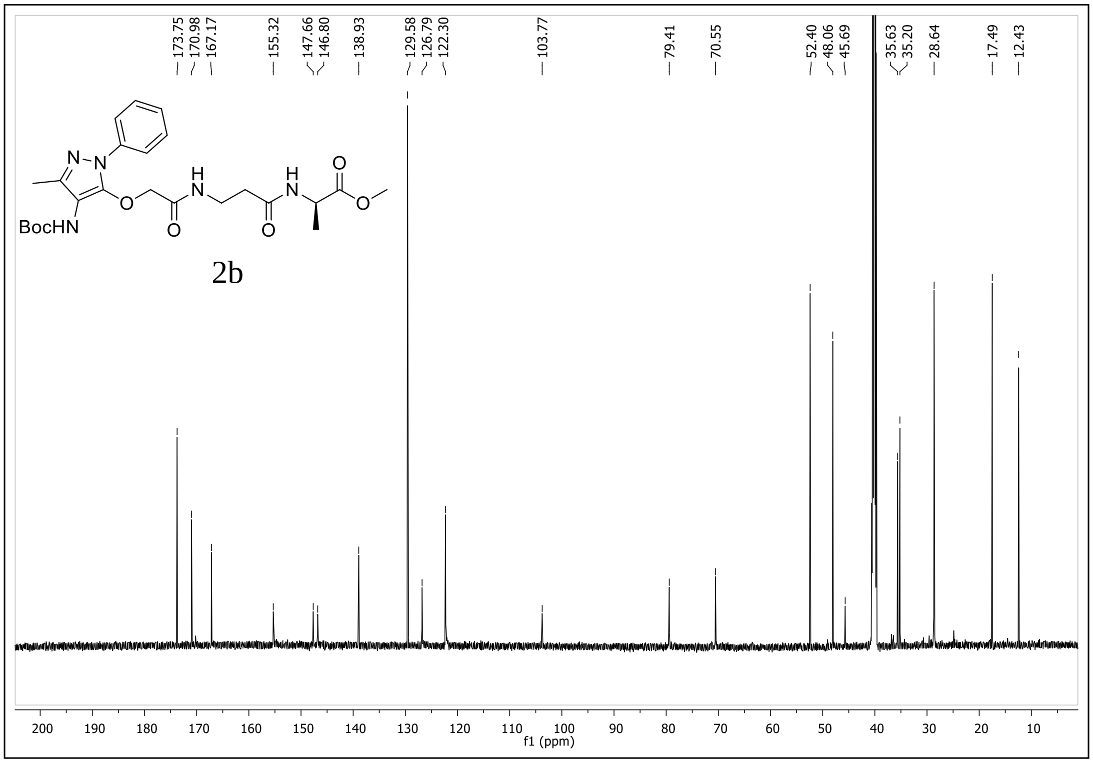

Figure S3. ^{1}H-NMR (400MHz) and ^{13}C-NMR (176MHz) spectra of **2b** in DMSO-d6

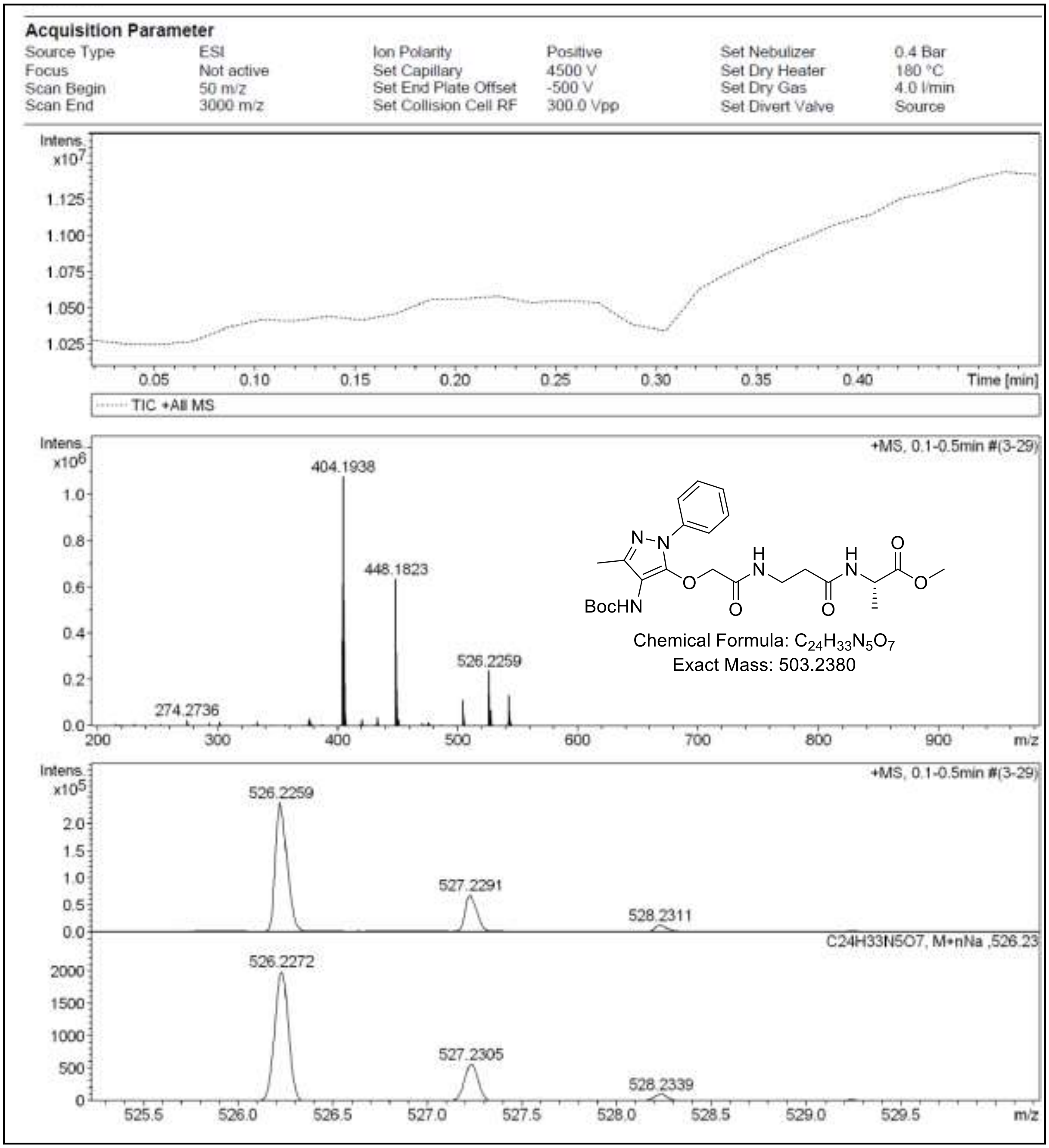

Figure S4. ESI-MS/HRMS spectra of **2b**

3. ^{1}H-/^{13}C-/ ESI-MS/HRMS spectra of **2c**

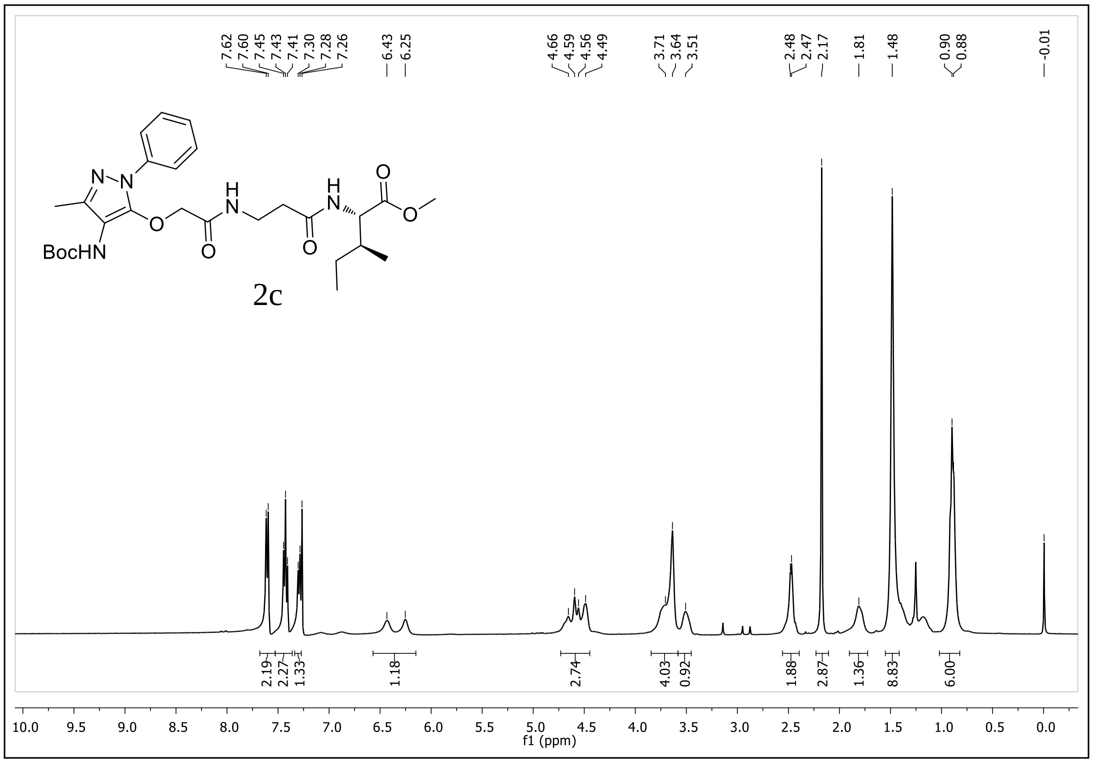

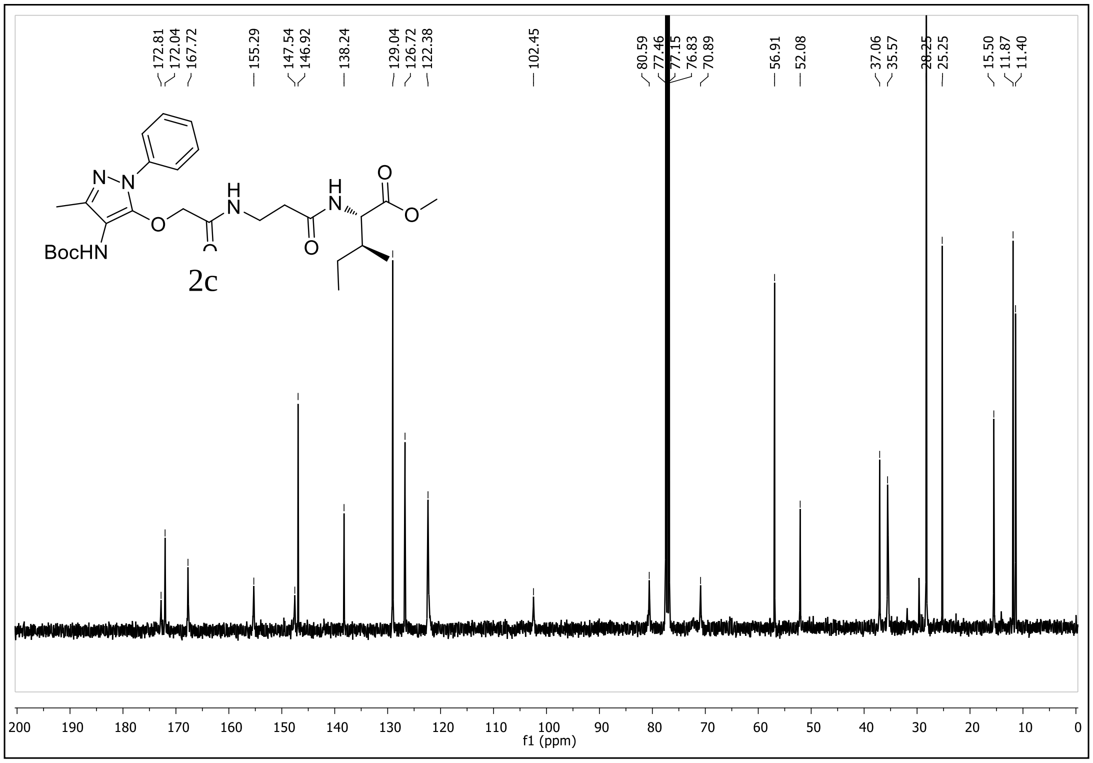

Figure S5. ^{1}H-NMR (400MHz) and ^{13}C-NMR (176MHz) spectra of **2c** in $CDCl_3$

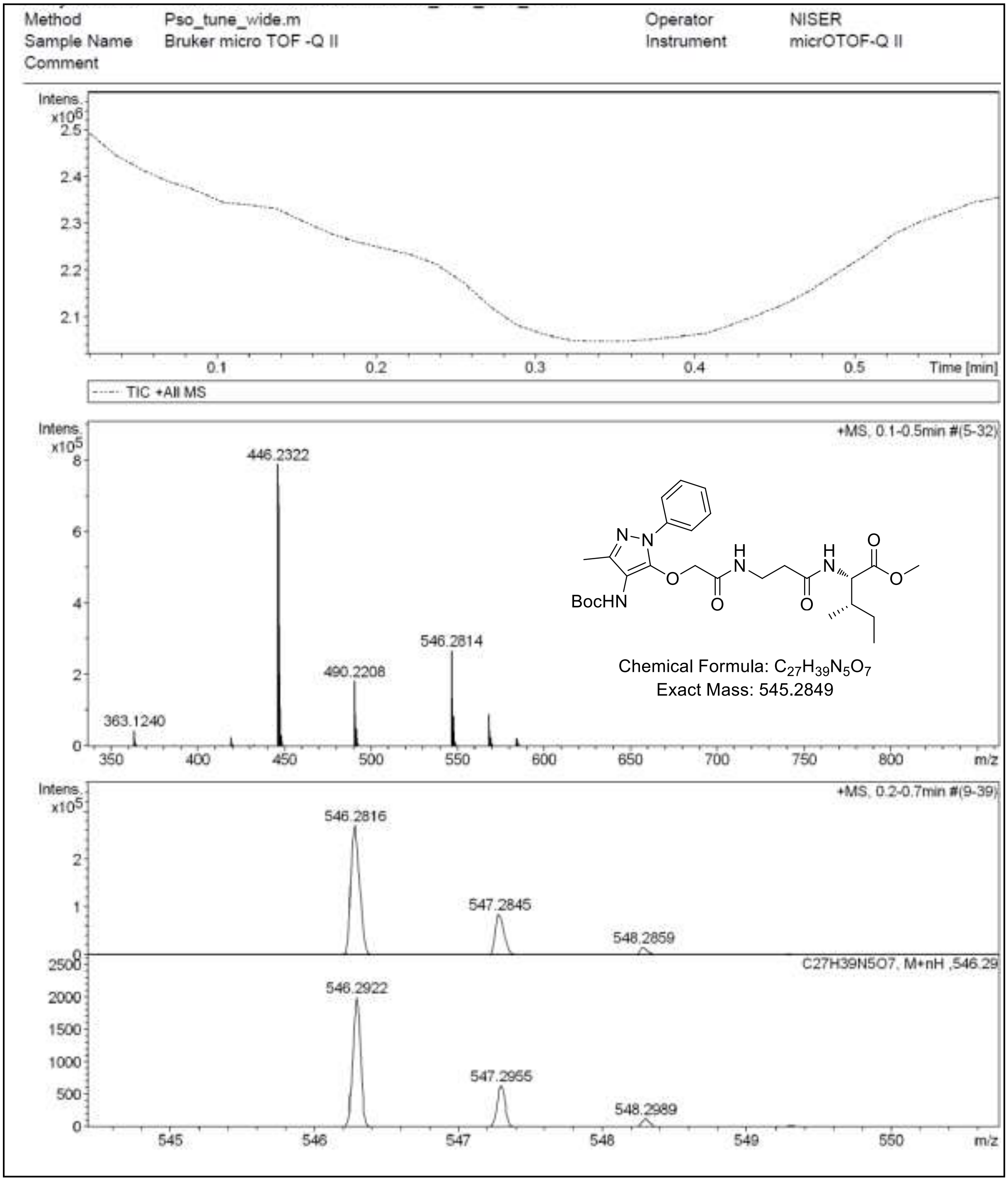

Figure S6. ESI-MS/HRMS spectra of **2c**

4. ^{1}H-/^{13}C-/ ESI-MS/HRMS spectra of **2d**

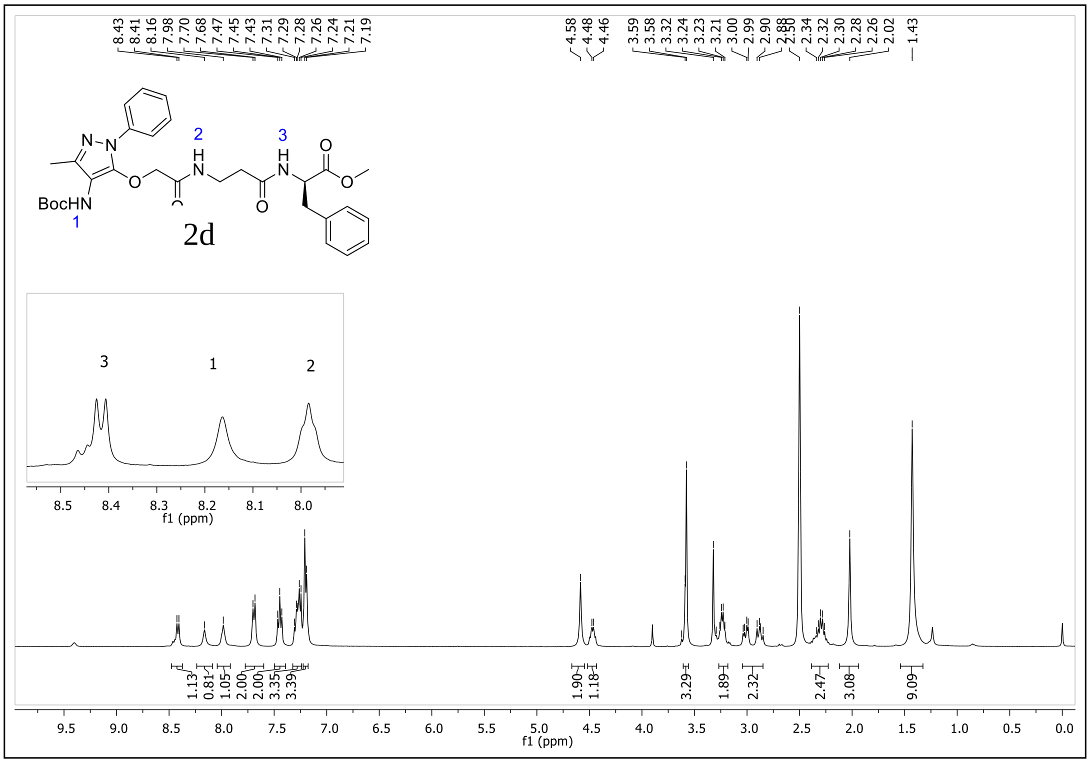

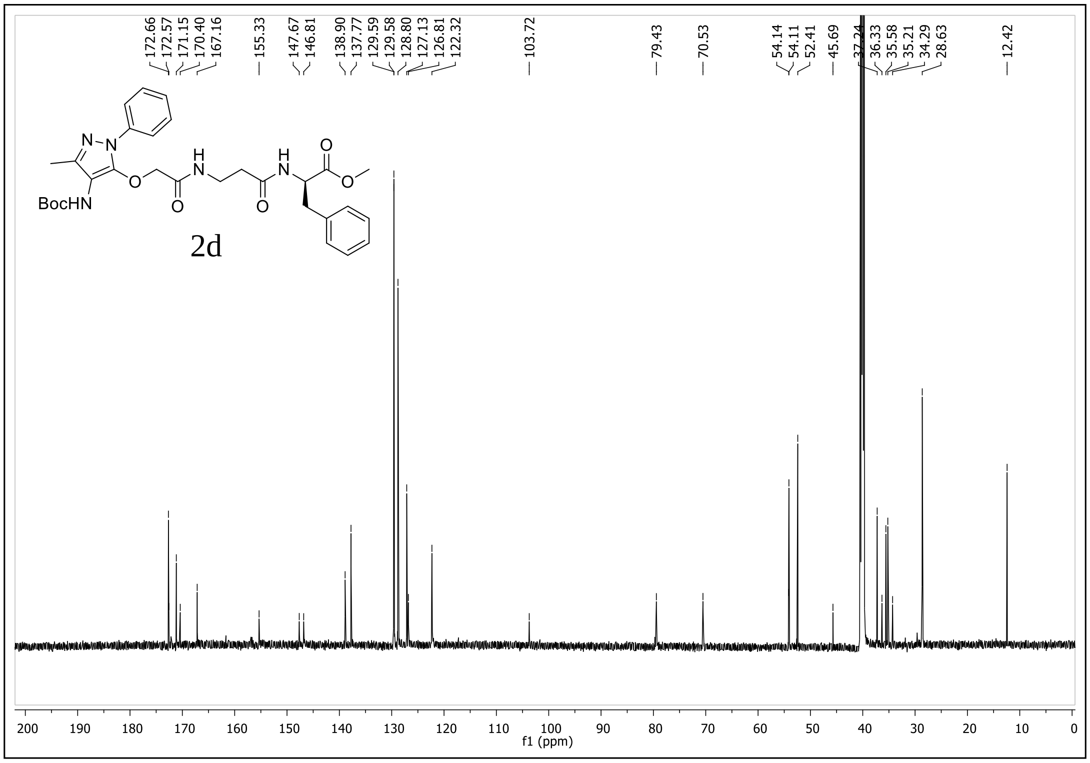

Figure S7. ^{1}H-NMR (400MHz) and ^{13}C-NMR (176MHz) NMR spectra of **2d** in DMSO-d6

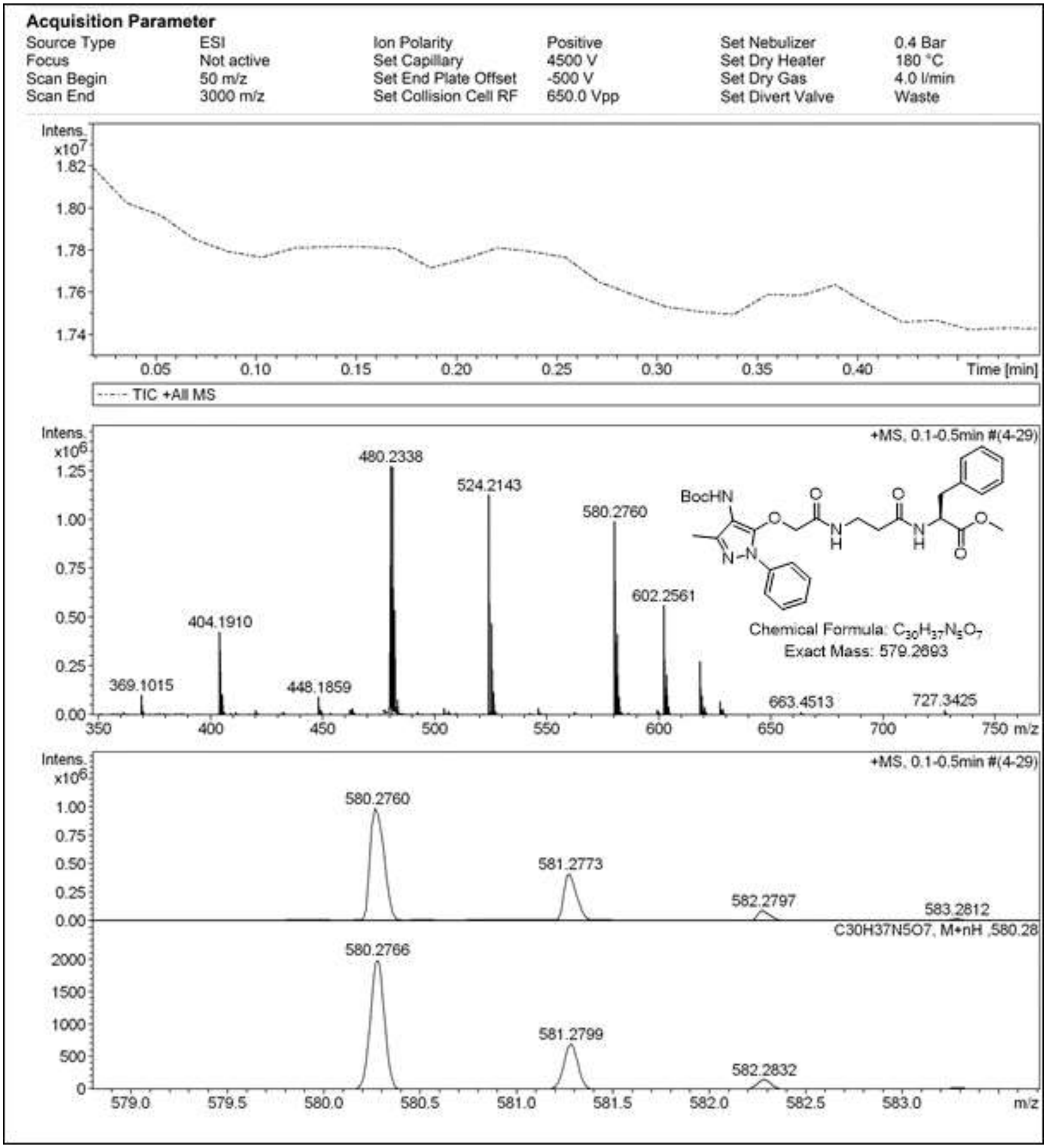

Figure S8. ESI-MS/HRMS spectra of **2d**

5. ^{1}H-/^{13}C-/ ESI-MS/HRMS spectra of **2e**

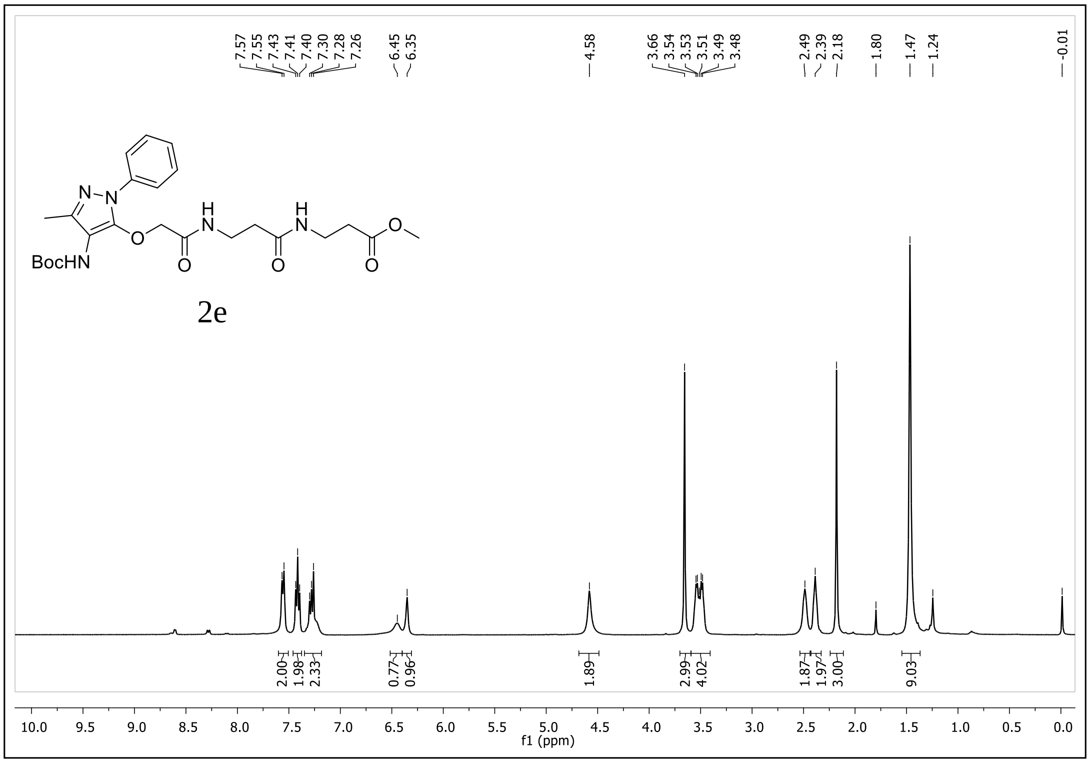

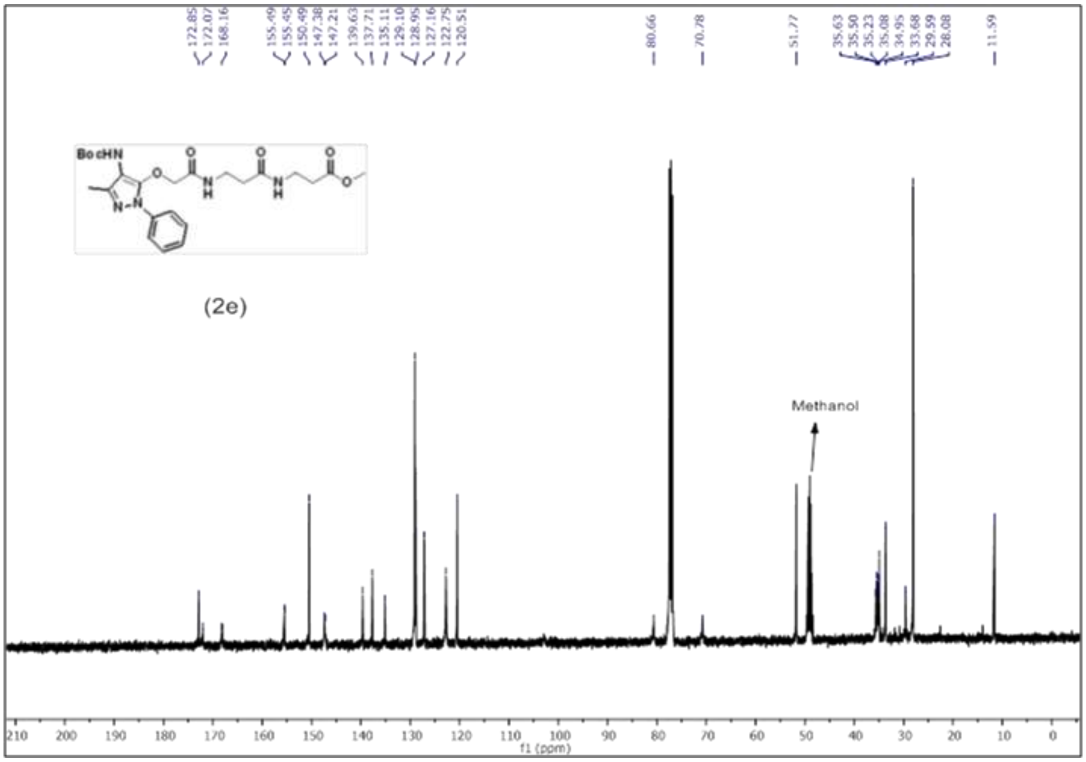

Figure S9. ^{1}H-NMR (400MHz) and ^{13}C-NMR (176MHz) spectra of **2e** in $CDCl_3$ (1 drop Methanol-D_4)

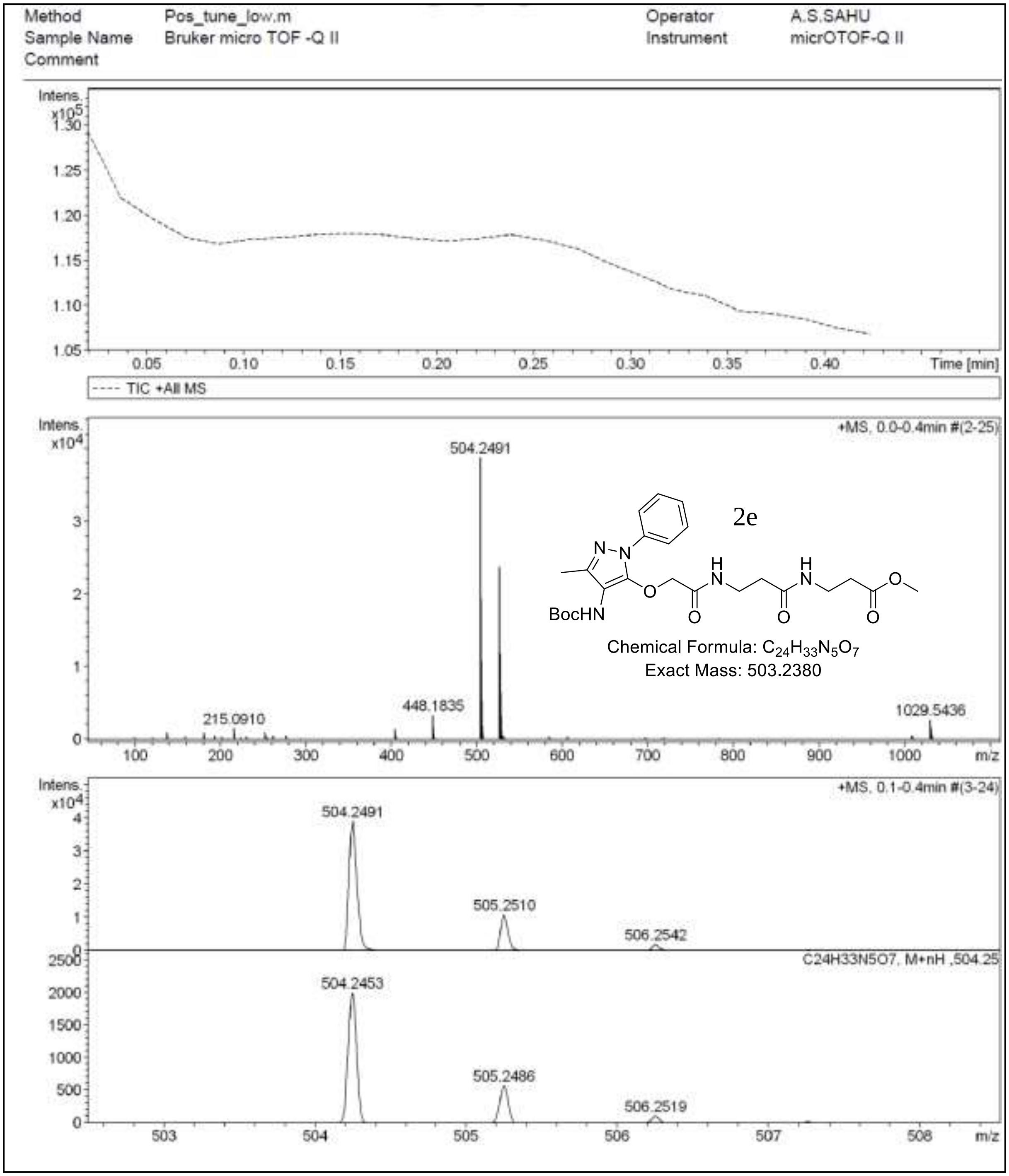

Figure S10. ESI-MS/HRMS spectra of **2e**

6. Circular Dichroism (CD) spectra control peptides and **2a-2e**.

CD spectra were recorded in degassed CH_3OH, AcCN, $CHCl_3$, and CF_3CH_2OH at 20 ^{0}C from 300-190 nm with peptide concentrations of 0.1 mM. CD data is collected with following parameters, Data pitch 2 nm, DIT 2 sec, bandwidth 2 nm, scanning speed 100 nm/min.

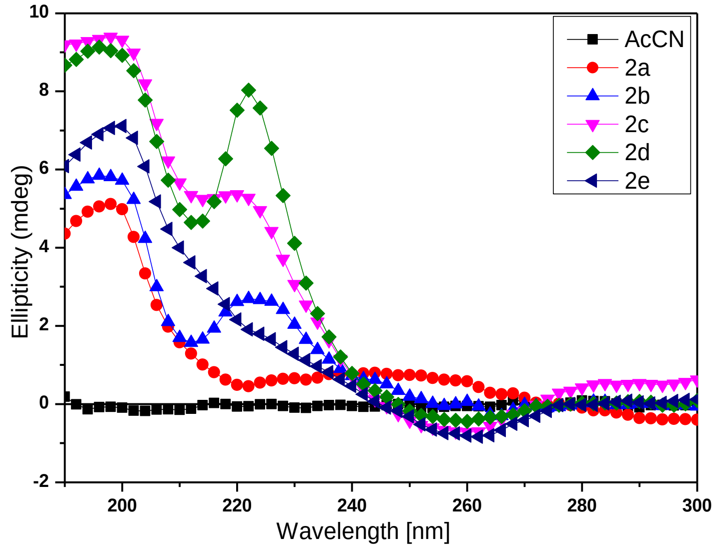

Figure S11. CD spectra of peptide **2a-2e** in Acetonitrile (AcCN)

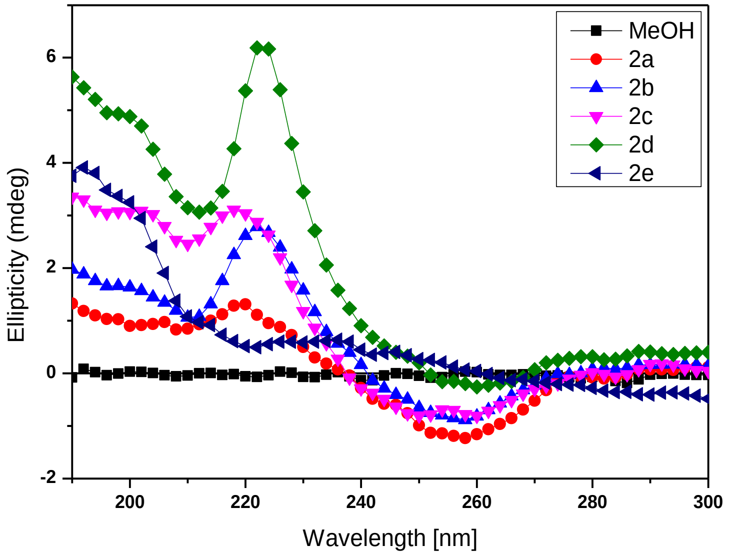

Figure S12. CD spectra of peptide **2a-2e** in Methanol (MeOH)

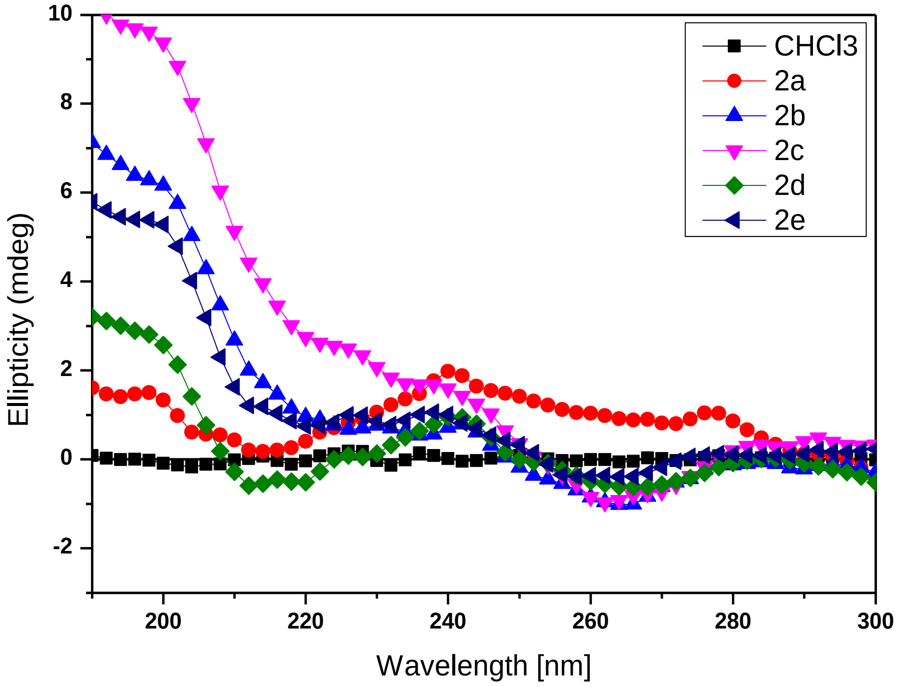

Figure S13. CD spectra of peptide **2a-2e** in Chloroform ($CHCl_3$)

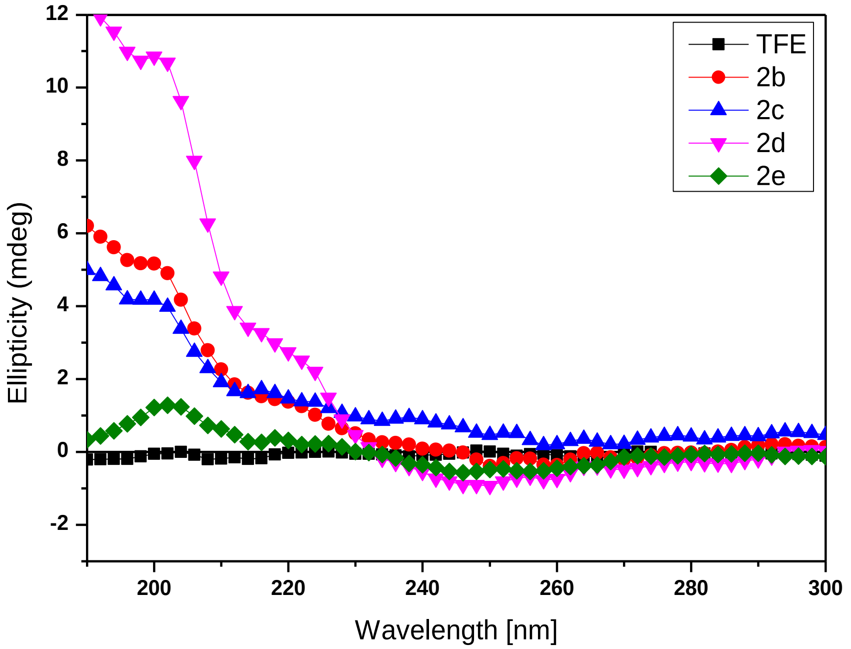

Figure S14. CD spectra of peptide **2a-2e** in Trifluoroethanol (TFE)

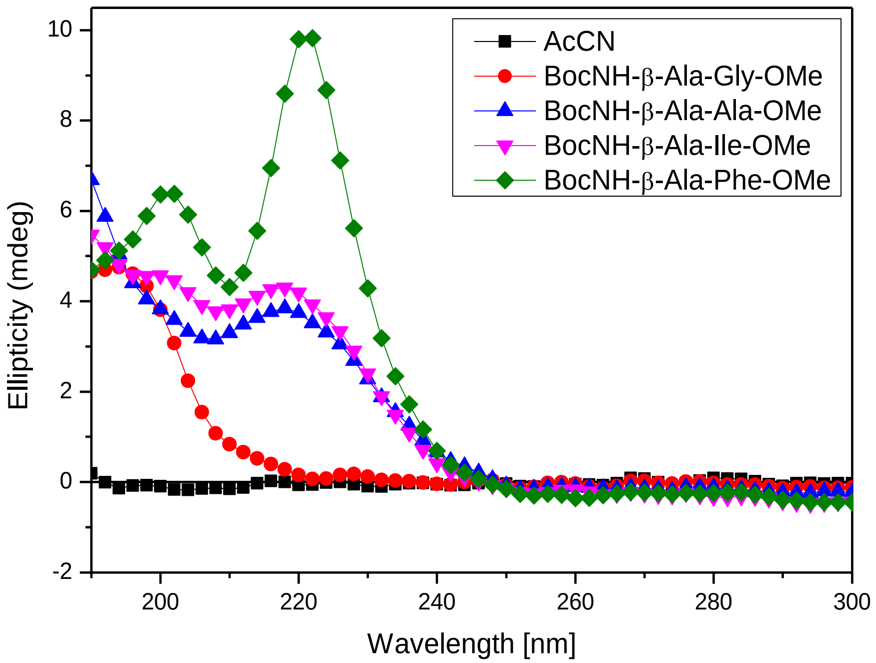

Figure S15. CD spectra of control peptides in Acetonitrile (AcCN)

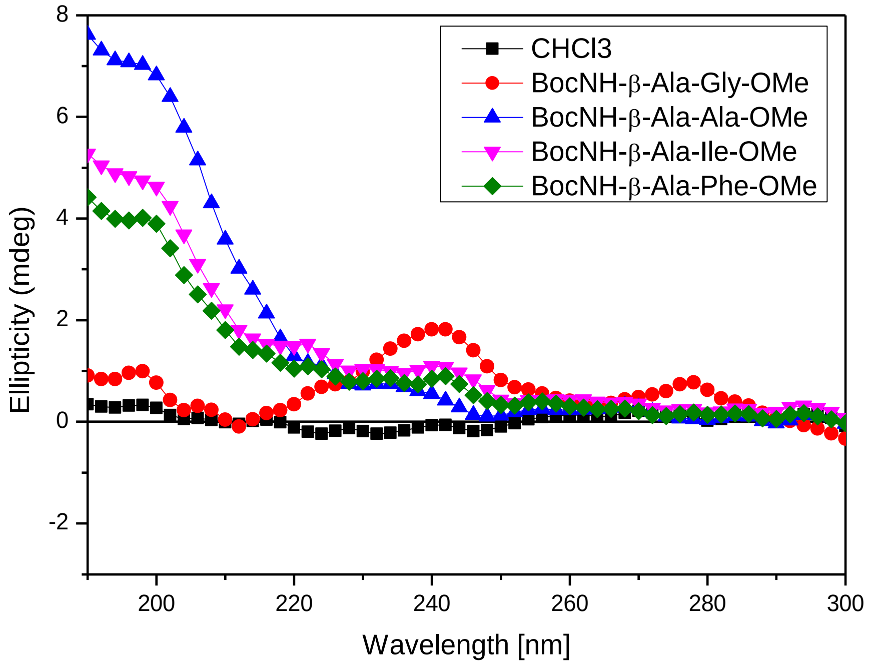

Figure S16. CD spectra of control peptides in Chloroform ($CHCl_3$)

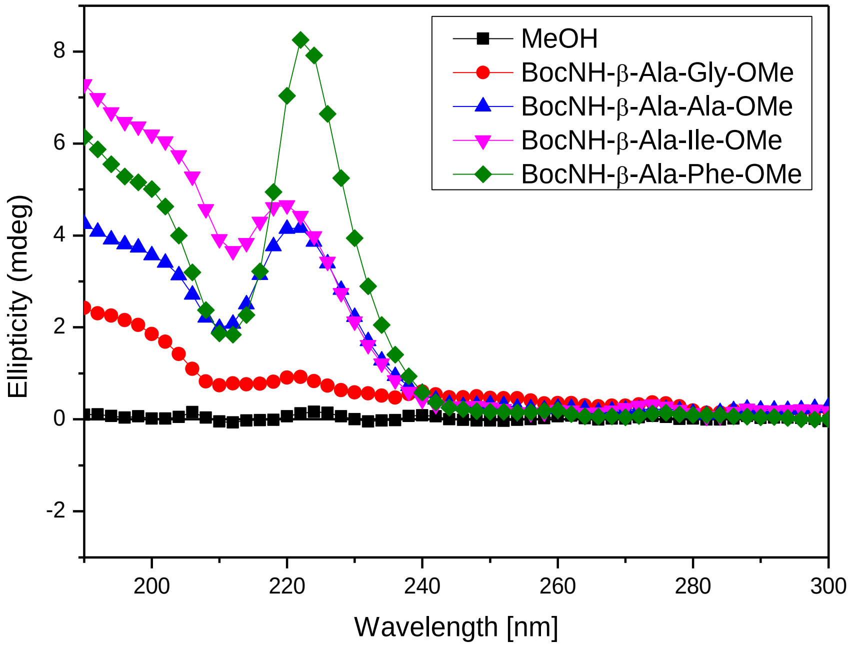

Figure S17. CD spectra of control peptides in Methanol (MeOH)

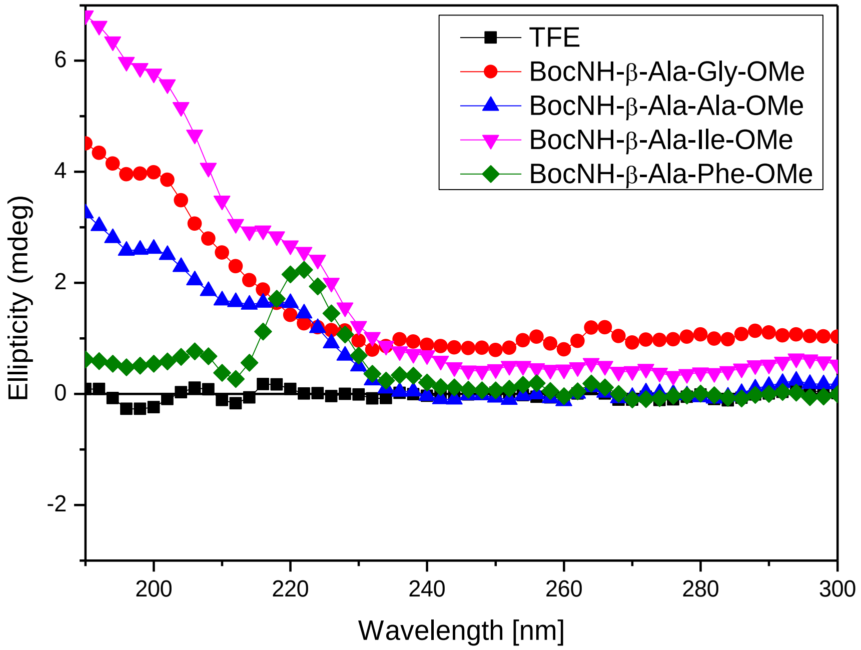

Figure S18. CD spectra of control peptides in Trifluoroethanol (TFE)

7. FT-IR spectra of peptide organogel (**2b/2c/2d/2e**)

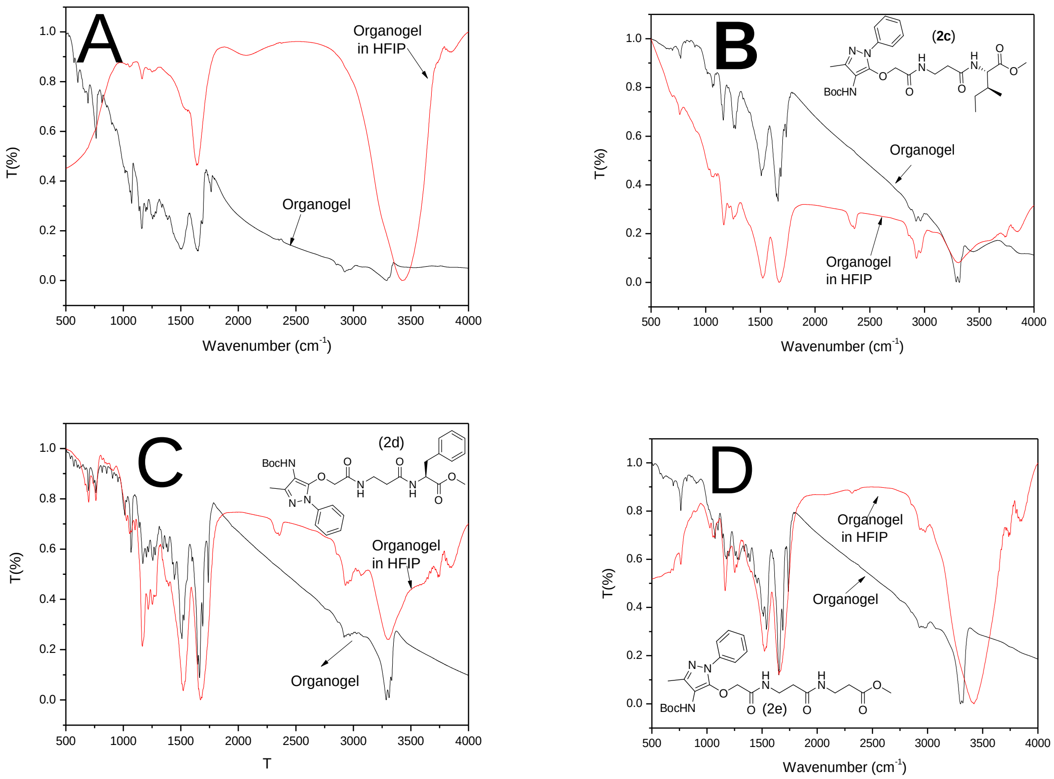

Figure S19. FT-IR spectra of organogel (neat) and HFIP for APA peptides, **2b** (A), **2c** (B), **2d** (C), and **2e** (D).

8. SEM Images of peptide organogel (**2b/2c/2d/2e**)

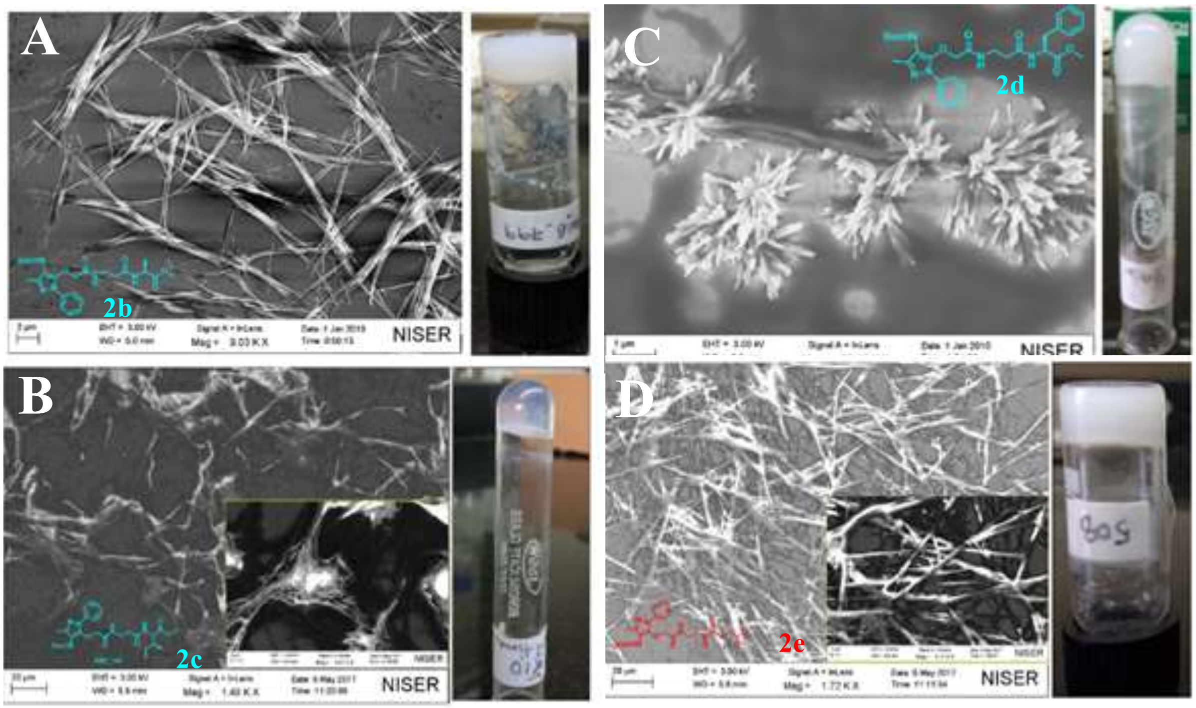

Figure S20. Aminopyrazolonylated α/β-hybrid peptide organogels. SEM-images and organogels in inverted test tube: (A) peptide **2b**; (B) Peptide **2c**; (C) Peptide **2d**; and (D) peptide **2e.**

9. Powder XRD of peptides **2b/2c/2d/2e.**

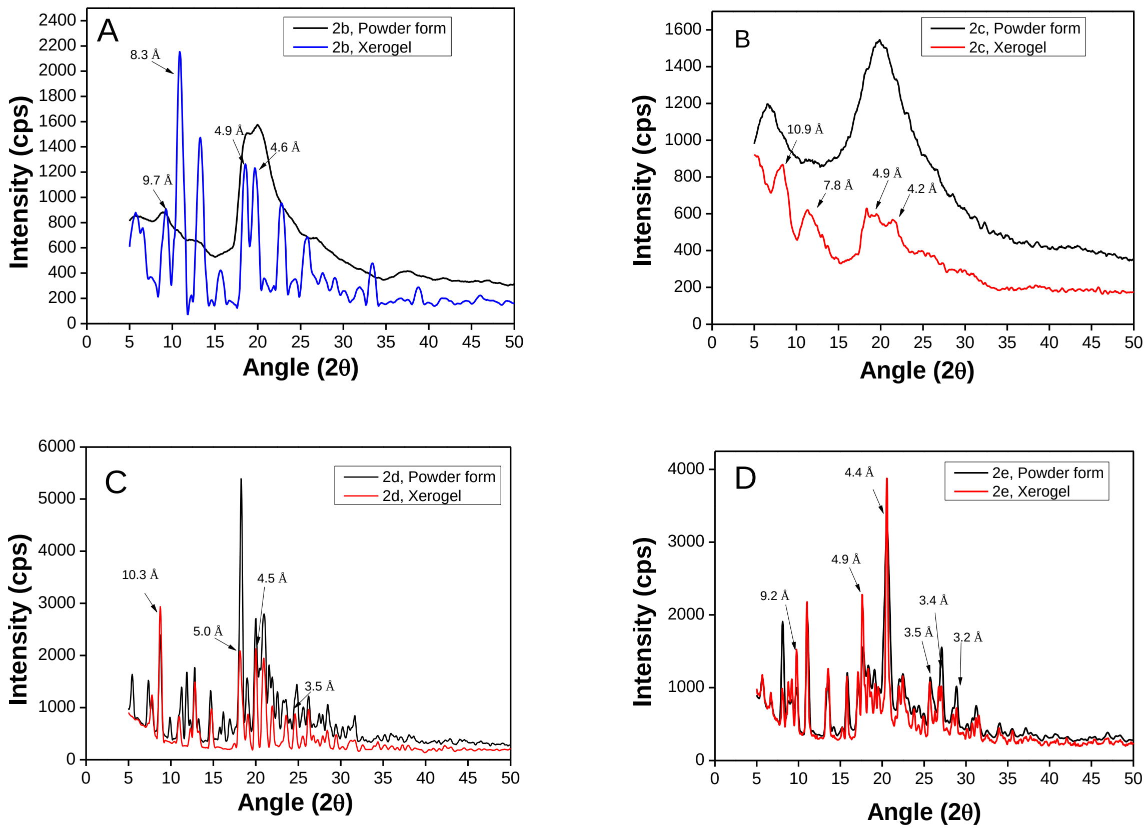

Figure S21. Powder XRD plot of powder & xerogel forms of APA peptides **2b** (A), **2c** (B), **2d** (C) **2e** (D).

10. ^{1}H-COSY- NMR and DMSO-d6 titration

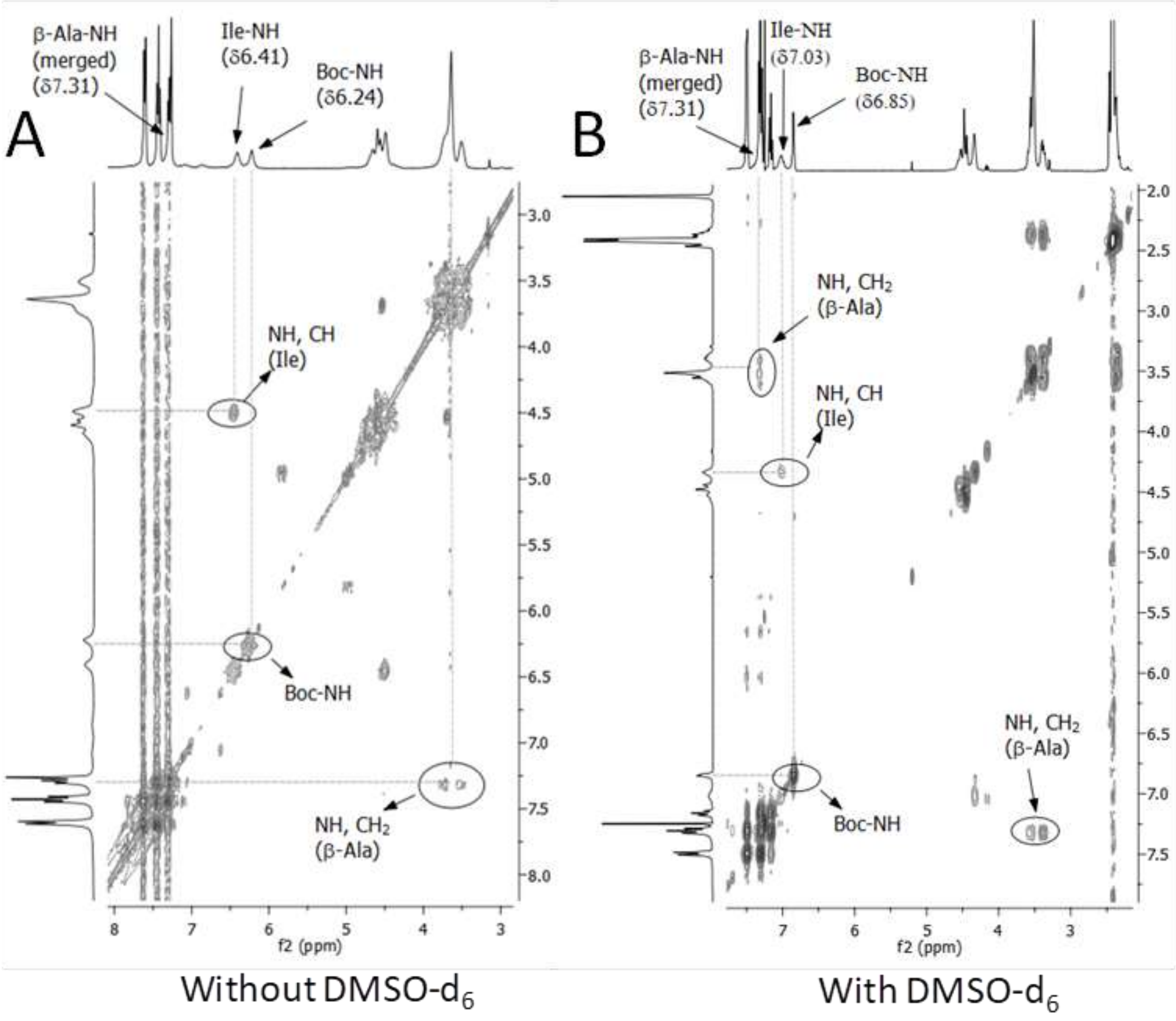

Figure S22. ^{1}H-COSY spectra of APA-peptide, **2c** in $CDCl_3$. Without DMSO-d6 (A) and with 19 μl DMSO-d6 (B).

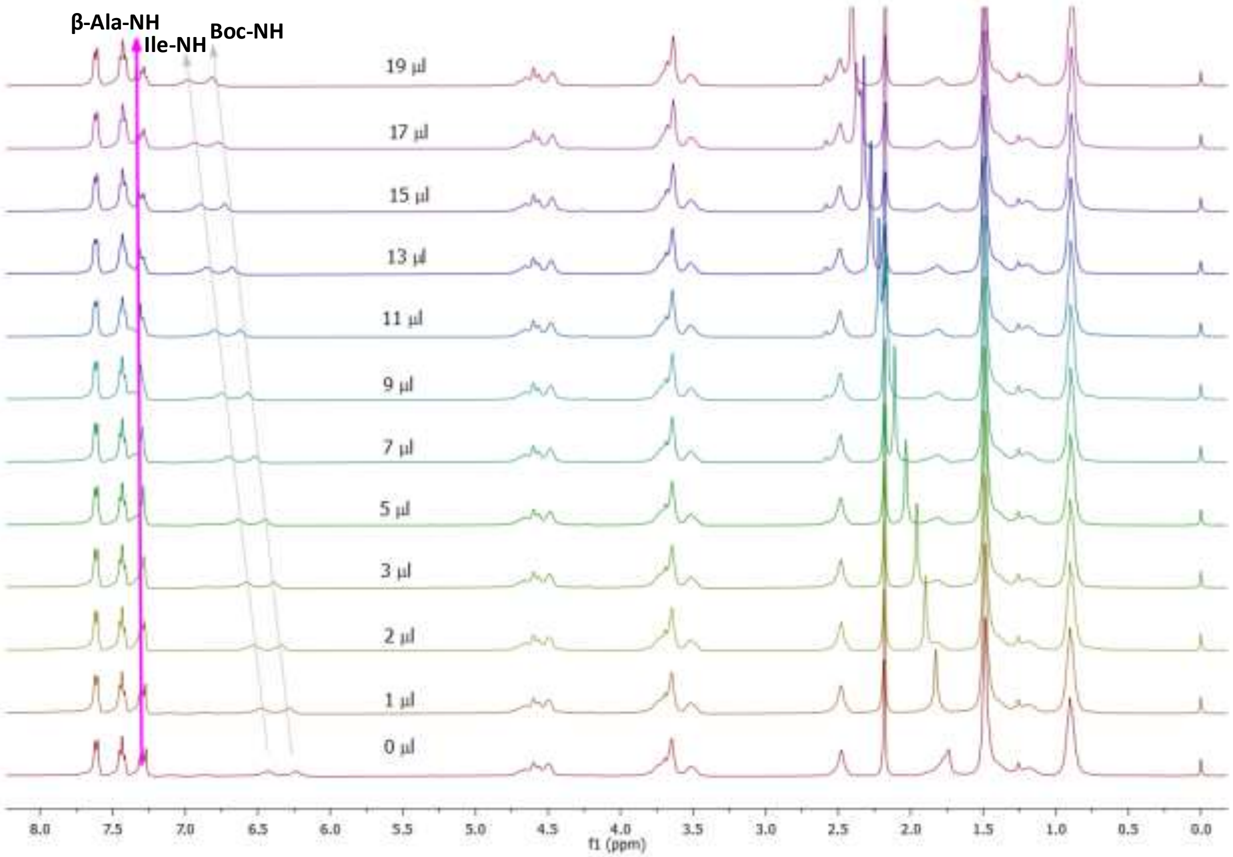

Figure S23. ^{1}H-NMR of DMSO-d6 titration of APA peptide **2c** in $CDCl_3$.

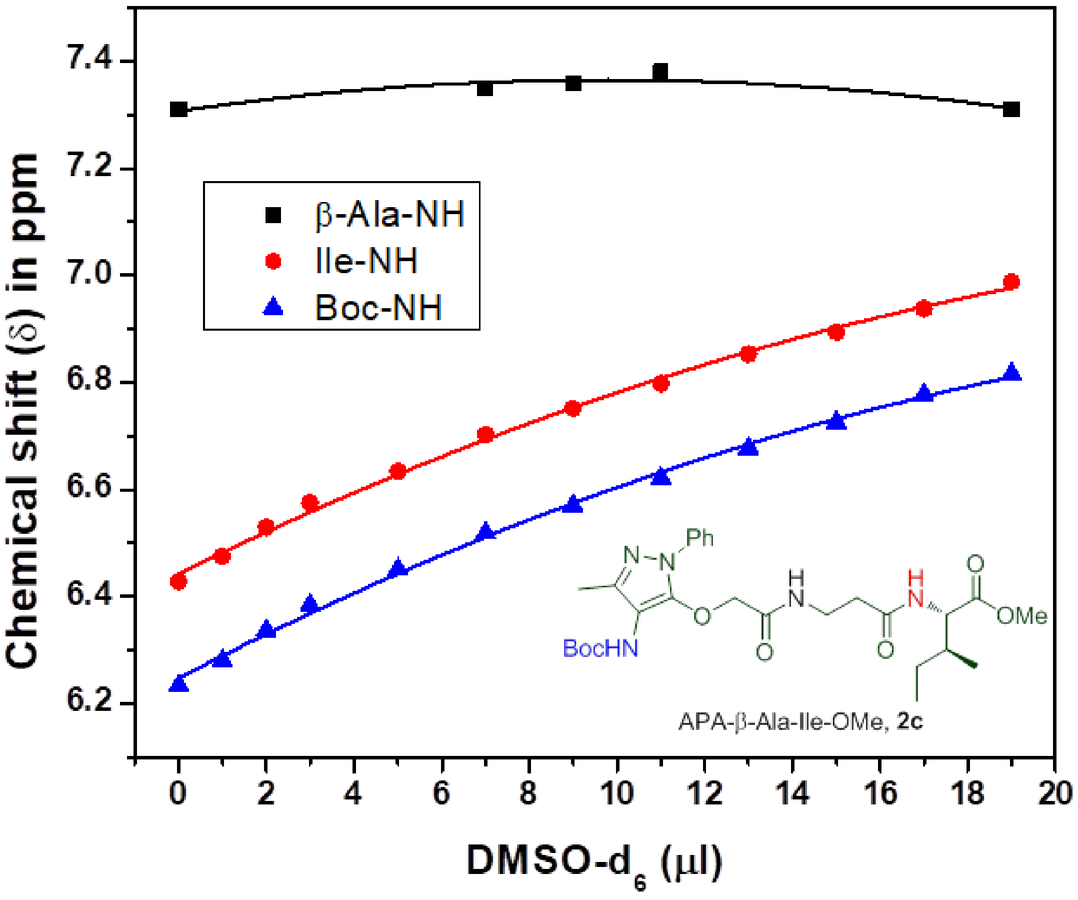

Figure S24. DMSO-d6 titration profile of amide NH in APA peptide **2c** in $CDCl_3$.

11. TGA and Derivative TGA plots of peptides **2b/2c/2d/2e.**

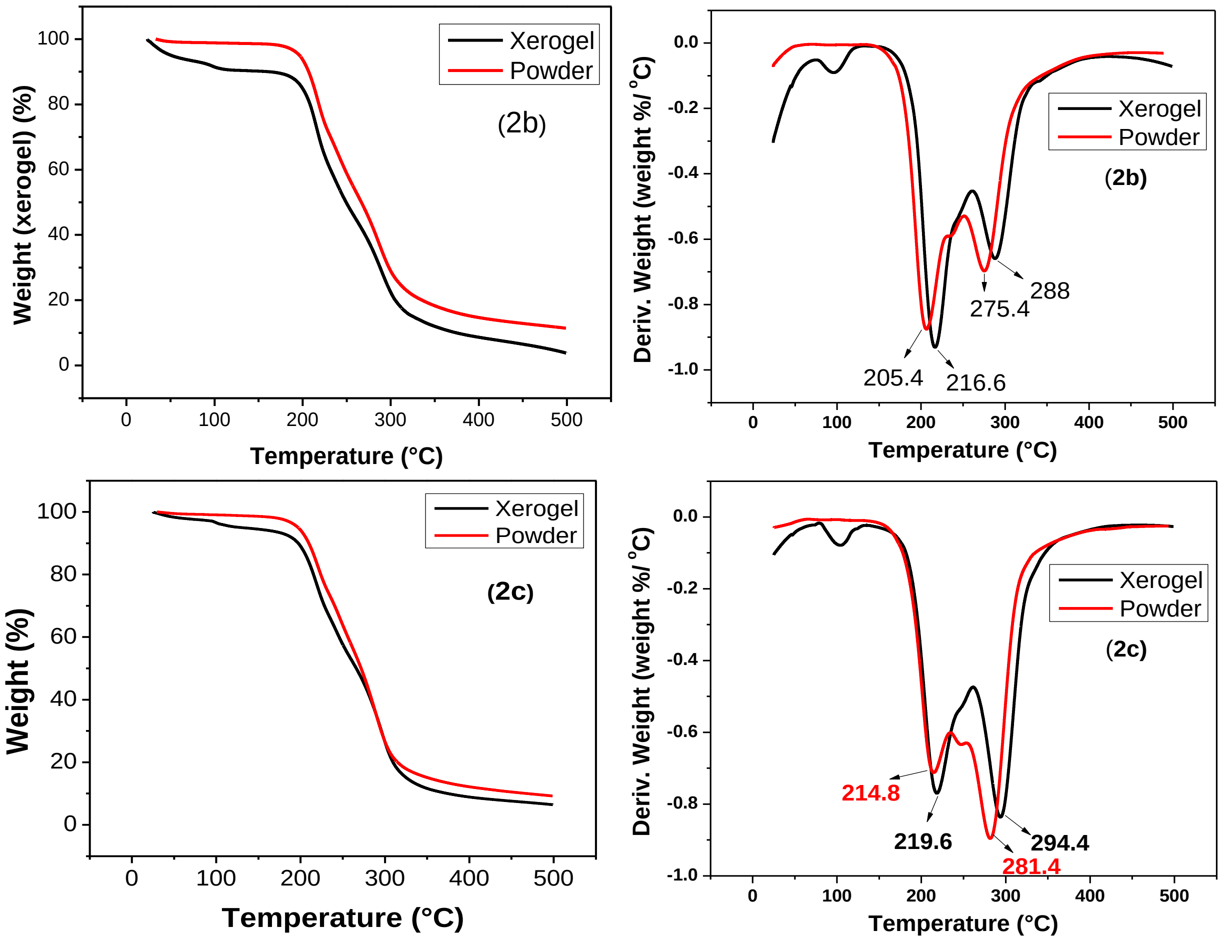

(cont.)

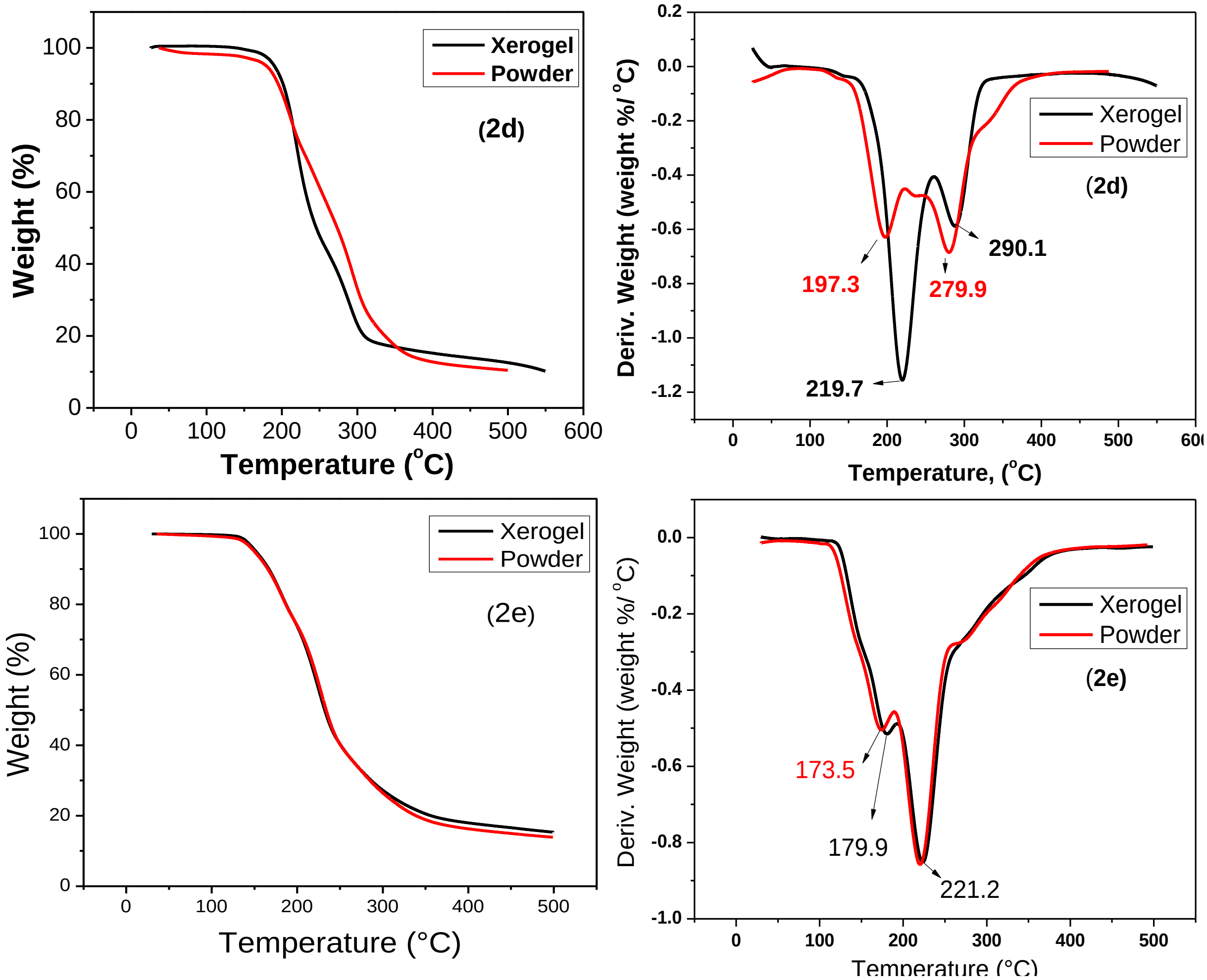

Figure S25. TGA (left panel) and Derivative TGA (right panel) plots of organogel & powder form of peptides (**2b**-**2e**).

12. UV-Vis Spectra of peptides **2c/2e in MeOH.**

We attempted to UV-vis spectra of peptides in ethyl acetate and hexane system but could not record owing to the precipitation. Thus we recorded the UV-spectra of representative peptides in MeOH.

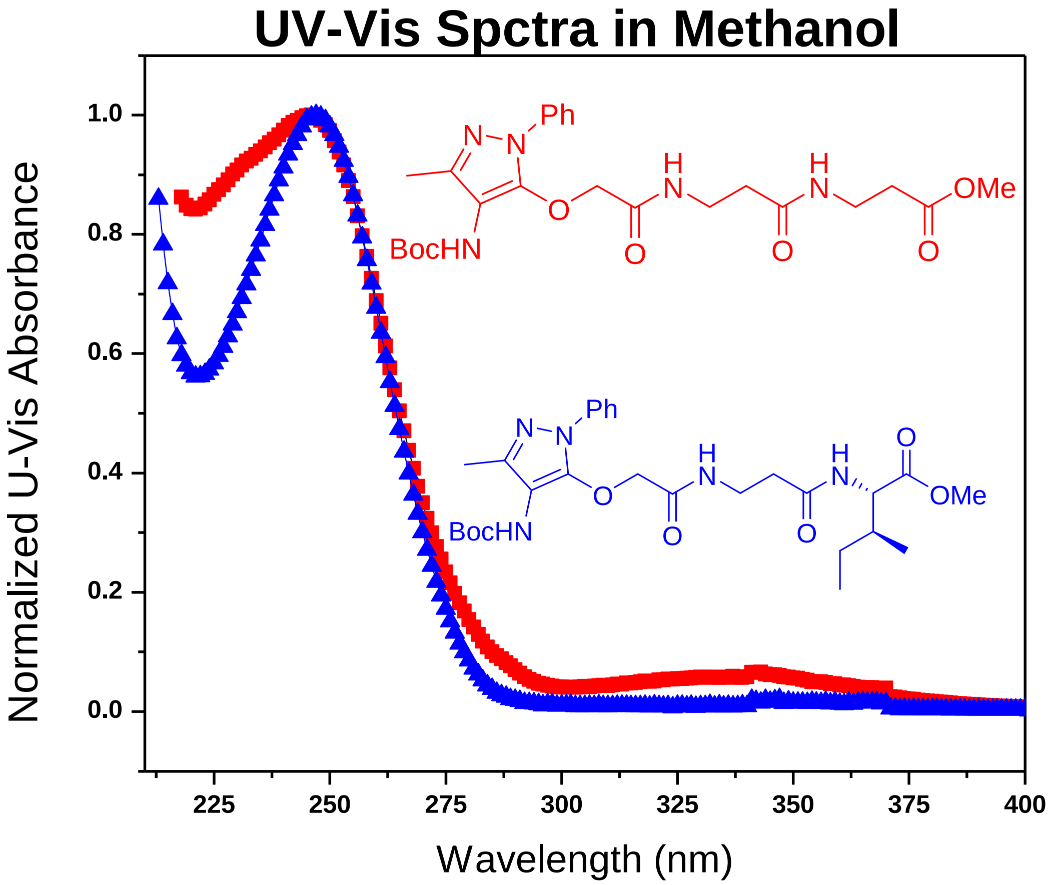

Figure S26. UV-Vis Spectra of APA-Peptides in MeOH

13. Theoretical calculation of APA-peptides conformers

GMMX and GBSA solvation model are versatile theoretical methods for energy minimized structural conformation of peptidomimatics.[1, 2]

Stepwise methods for the calculation of energy minimized structure: **Step-1**: ChemDraw (*.mol); **Step-2**: Gauss view 6.1.1 (GMMX conformer calculation; conformer search, Force field MMFF94; Enegry windo 3.5 (Kcal/mol; Max. serach 10000);; **Step-3**: GBSA solvation model; Pcm10 software; conditions: Force field MMFF94; solvent dielectric 78.3 and internal dielectric: 1; **Step 4**: Image from gauss view 6.1.1.

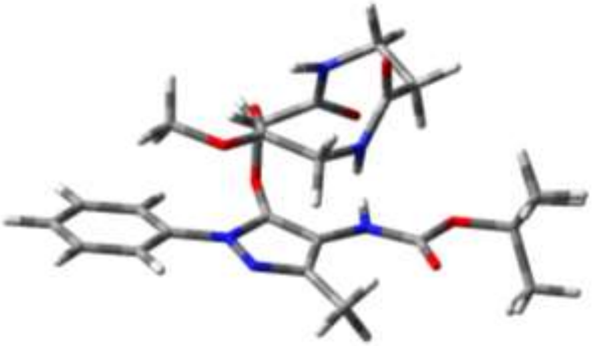

2a

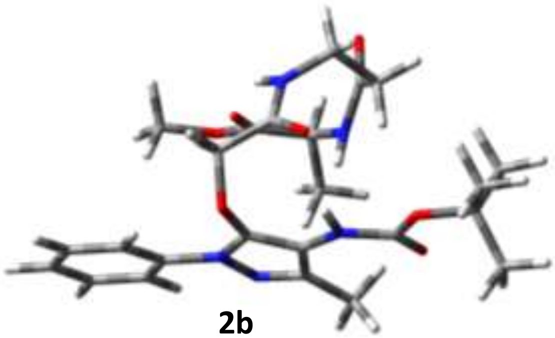

2b

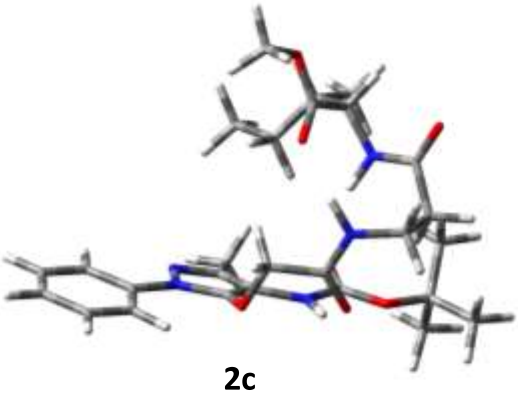

2c

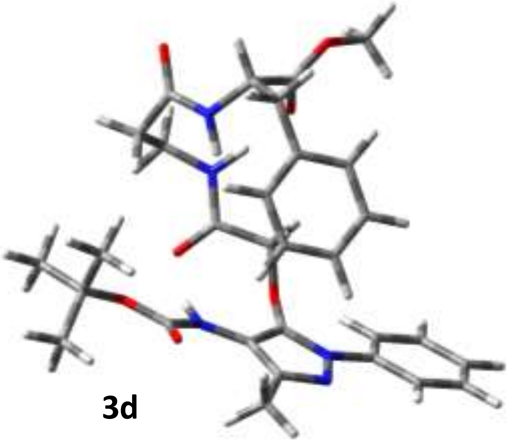

3d

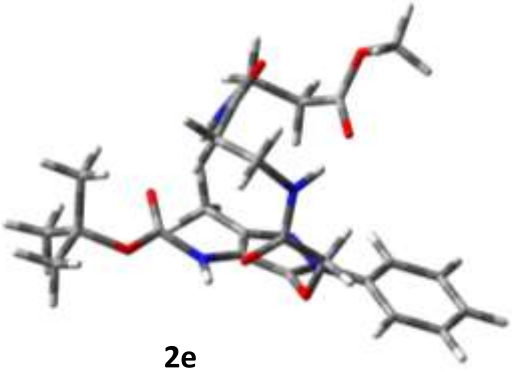

2e

Figure S27. Energy mimimized conformer with H-atoms: **2a** (E = 31.2kcal/mol; GBSA Steric Energy = 18.6 kca/mol; Dielectric constant: 1; Dipole moment: 4.2); **3b** (E = 34.7kcal/mol; GBSA Steric Energy = 22.3kca/mol; Dielectric constant: 1; Dipole moment: 4.2); **3c** (E = 38.8kcal/mol; GBSA Steric Energy = 25.8kcal/mol; Dielectric constant: 1; Dipole moment: 5.0); **3d** (E = 56.0kcal/mol; GBSA Steric Energy = 41.4kcal/mol; Dielectric constant: 1; Dipole moment: 6.0); **2e** (E = -3.0kca/mol; GBSA Steric Energy = -15.6 kca/mol; Dielectric constant: 1; Dipole moment: 3.1); GBSA: Generalized Born Surface Area

14. Reference

1. Biswas, S.; Abo-Dya, N. E.; Oliferenko, A.; Khiabani, A.; Steel, P. J.; Alamry, K. A.; Katritzky, A. R., Oxyazapeptides: A New Peptidomimetics Family. Synthesis, Structure Determination and Conformational Analysis. *Journal of Organic Chemistry*. **2013**, 78, 8502−8509
2. Lee, H.-J.; Choi, K.-H.; Ahn, I.-A.; Ro, S.; Jang, H. G.; Choi, Y.-S.; Lee, K.-B., The β-turn preferential solution conformation of a tetrapeptide containing an azaamino acid residue. *Journal of Molecular Structure* **2001,** *569* (1-3), 43-54.
